# Effect of Amorphous Silicates on the Neutralization of Tricalcium Aluminate Hexahydrate Relevant to Bauxite Residue Treatment

**DOI:** 10.1021/acs.inorgchem.6c00005

**Published:** 2026-02-23

**Authors:** Yvette Szabó, Meerab Asher, Réka Zahorán, Judit Papp, Dániel Sebők, Pál Sipos, Márton Szabados, Markus Gräfe, Bence Kutus

**Affiliations:** a Department of Molecular and Analytical Chemistry, 37442 University of Szeged, Dóm tér 7−8, Szeged H−6720, Hungary; b Department of Applied and Environmental Chemistry, 37442 University of Szeged, Rerrich Béla tér 1, Szeged H−6720, Hungary; c Bauxite Residue R&D Group, Technology Development & Transfer, Emirates Global Aluminum, PO Box 3627 Dubai, United Arab Emirates

## Abstract

Acid neutralization is an efficient way to lower the solution pH of bauxite residue slurries (generated via the Bayer process) to a level that enables safe handling and further utilization. Tricalcium aluminate hexahydrate (TCA, Ca_3_Al_2_(OH)_12_) and its silicate-substituted polymorph, katoite (KAT, Ca_3_Al_2_(SiO_4_)*_*x*_* (OH)_12–4*x*_), are one of the major sources that contribute to the high alkalinity of residues. Yet, the neutralization chemistry of KAT phases is poorly understood. To this end, we synthesized TCA and KAT and studied their acid–base reactions. We find the as-prepared pseudo-KAT (PKAT) phase to be a poorly substituted TCA with *x* ≈ 0.05 while containing amorphous calcium silicate hydrate as well as sodium aluminosilicate minor phases. Upon addition of HCl, TCA first transforms to Friedel’s salt, a layered double hydroxide hosting Cl^–^ ions, which is absent for PKAT. Further, the lack of LDH precipitation, closely related to calcium silicate hydrate, gives rise to a lower buffering range (by ∼0.5–1.2 pH units) for PKAT. Another striking consequence of minor silicate phases in PKAT is the ∼17% smaller acid consumption as compared to TCA. In conclusion, amorphous silicates markedly affect both the neutralization mechanism and capacity of tricalcium aluminate hydrates.

## Graphical abstract

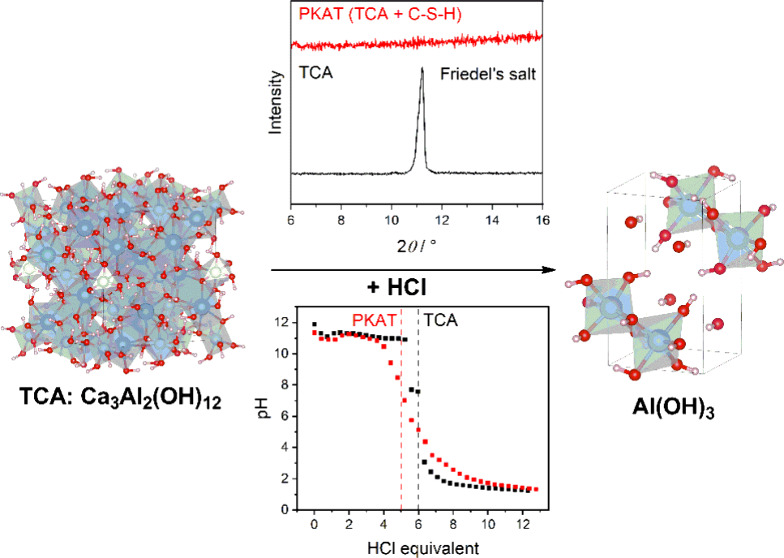

## Introduction

Bauxite residue or BxR is the byproduct of the Bayer process, in which aluminum-rich bauxite is digested in hot, concentrated NaOH solution followed by precipitation and calcination to obtain pure alumina.^1,2^ Based on the estimated global aluminum production of 146.7 million tons^3^ and an average residue output of 1.5 tons per ton of alumina,^1,4,5^ ca. 220 million tons of BxR have been generated in 2024, rendering this byproduct one of the major industrial wastes globally. As a result of its strongly alkaline pH, ranging between 9.2 and 12.8 with an average value of 11.3,^1^ BxR is an environmental hazard, necessitating the development of efficient treatment technologies that can permanently bring down the pH of effluents to <9.^6^ This could not only reduce the risks and costs of BxR deposition and monitoring of existing impoundments,^1^ but could also facilitate residue valorization as soil,^1,4,6,7^ cement additive,^4,6^ or adsorbent.^6,8^

BxR neutralization^1,2,5,6^ is a plausible but not straightforward avenue to circumvent the issues associated with its high alkalinity. Residual process liquor is composed mainly of NaOH, NaAl­(OH)_4_, and Na_2_CO_3_,^1,5,9^ which can be readily neutralized, and such practice is already adopted by several alumina plants before discharging BxR.^1^ However, several solid phases forming during the Bayer process undergo slow (and partial) dissolution in aqueous medium, releasing OH^–^ ions into solution.^1,2,5,9^ Considering an acid–base titration curve of BxR, such reactive solids are associated with a specific pH buffer region determined by their solubility product.^1,10^ As a result, the pH slowly rebounds to ∼10 in leachates of various BxR deposits even after acidic treatment.^1,2,5,11^ Thus, complete neutralization of BxR with stable pH values below 9 is indispensable to mitigate environmental impacts and requires an understanding of the neutralization chemistry of sparingly soluble solids present in BxR.

Tricalcium aluminate hexahydrate, Ca_3_Al_2_(OH)_12_, or TCA, is one of the major sources of alkalinity in BxR,^1,2,5,9^ represented by the following dissolution reaction:^12^

$${\mathrm{Ca}}_{3}{\mathrm{Al}}_{2}{\left(\mathrm{OH}\right)}_{12}⇌3{\mathrm{Ca}}^{2+}+2\mathrm{Al}{{\left(\mathrm{OH}\right)}_{4}}^{-}+4{\mathrm{OH}}^{-}$$ 1

with log *K*_sp_ = −20.50 (*K*_sp_ being the solubility product),^12^ giving rise to pH = 11.83, assuming an infinitely dilute (ideal) solution saturated with TCA. TCA may amount to 20 wt % in BxR,^13^ and is commonly formed via the reaction between sodium aluminate and slaked lime (Ca­(OH)_2_), the latter being used to reduce soda loss prior to digestion, to facilitate the extraction of alumina, and to recover NaOH from Na_2_CO_3_ postdigestion.^1,2,9,14–16^ In our previous work,^17^ we studied the neutralization reactions between TCA and hydrochloric acid in the pH range of 1–12.5. First, TCA transforms completely to Friedel’s salt, a layered double hydroxide accommodating Cl^–^ ions and water molecules in the interlayer space:

$${\mathrm{Ca}}_{3}{\mathrm{Al}}_{2}{\left(\mathrm{OH}\right)}_{12}+2H^{+}+{\mathrm{Cl}}^{-}⇌\frac{1}{2}{\mathrm{Ca}}_{4}{\left[\mathrm{Al}{\left(\mathrm{OH}\right)}_{6}\right]}_{2}{\mathrm{Cl}}_{2}\cdot 4H_{2}O+{\mathrm{Ca}}^{2+}+\mathrm{Al}{{\left(\mathrm{OH}\right)}_{4}}^{-}$$ 2

Further addition of acid leads to the dissolution of the LDH, accompanied by the precipitation of Al­(OH)_3_ at pH ≈ 8. The complete neutralization of TCA requires 6 mol of H^+^ ions per mole of solid, i.e., 6 equiv of HCl:

$${\mathrm{Ca}}_{3}{\mathrm{Al}}_{2}{\left(\mathrm{OH}\right)}_{12}+6H^{+}⇌3{\mathrm{Ca}}^{2+}+2\mathrm{Al}{\left(\mathrm{OH}\right)}_{3}+6H_{2}O$$ 3

TCA can be considered as the Si-free end member of silica-containing hydrogrossulars with a general composition of Ca_3_Al_2_(SiO_4_)*_*x*_* (OH)_12–4*x*_, where 4 OH^–^ ions are replaced by a SiO_4_^4–^ unit in an isomorphous manner,^18–20^ *x* representing the degree of substitution. As a result, all silicate-containing solids retain the cubic structure of TCA, but their cell constant decreases with increasing degrees of substitution, which in turn can be used to estimate the silica content in hydrogarnets.^18–22^ Further, mixtures of hydrogrossulars and TCA form solid solutions if the target value of *x* is smaller than 0.5 or higher than 1.5. In between, a miscibility gap occurs, and the solid separates into a silica-poor and silica-rich phase.^19,20^

In the context of BxR neutralization, formation of TCA is often accompanied or surpassed by the formation of low-silica substituted katoites (KAT), where *x* ≤ 1.5.^18^ The formation of KAT is most pronounced during the predesilication step, where solution-phase silica, arising from the alkaline dissolution of bauxitic kaolinite, reacts with NaAl­(OH)_4_ and Ca­(OH)_2_.^1,5,9,15^ In this respect, it has been shown that KATs can form under Bayer conditions with *x* ≈ 0.65 at 90 °C. This formation route is different from other hydrothermal methods published in literature, where CaO,^19^ Ca­(NO_3_)_2_,^20^ or tricalcium aluminate (Ca_3_Al_2_O_6_)^23^ was reacted with various silicon (SiO_2_, silicic acid, or Na_2_SiO_3_) and aluminum (Al_2_O_3_, NaAlO_2_, or AlCl_3_) sources.

The dissolution of KAT phases can be described with the following equation:^12^

$${\mathrm{Ca}}_{3}{\mathrm{Al}}_{2}{\left({\mathrm{SiO}}_{4}\right)}_{x}{\left(\mathrm{OH}\right)}_{12-4x}+3{xH}_{2}O⇌3{\mathrm{Ca}}^{2+}+2\mathrm{Al}{{\left(\mathrm{OH}\right)}_{4}}^{-}+x{H_{3}{\mathrm{SiO}}_{4}}^{-}+\left(4-x\right){\mathrm{OH}}^{-}$$ 4

with log *K*_sp_ and equilibrium pH values of −25.35 and 10.75 (*x* = 0.41), as well as −26.70 and 10.64 (*x* = 0.84).^12^ These values (as in the case of TCA) lie well above the target pH of <9 for BxR residues, rendering KAT an important source of alkalinity. Yet, no comprehensive study is available on the neutralization chemistry of this solid to the best of our knowledge.

To this end, we attempted to synthesize low-silica-substituted katoites with target degrees of substitution of *x* = 0.1–1 at room temperature, using TCA as a reactant. As a result, we obtained “pseudokatoites” (PKATs) whose diffraction pattern and composition differ only slightly from those of silica-free TCA. Further, PKAT with *x* = 1 contains a significant fraction of amorphous calcium silicate and sodium aluminosilicate. Strikingly, despite the crystalline fraction of PKAT being essentially TCA, its neutralization behavior is dramatically altered by the amorphous phases in terms of mechanism, buffering range, and acid consumption. Overall, this study sheds light on the crucial role of amorphous content (often encountered in BxR),^1^ on the acid–base reactions of TCA, and provides insights into the complex neutralization chemistry of BxR.

## Experimental Section

### Synthesis of TCA and Pseudokatoite, PKAT

As reference material, TCA was first prepared by hydrating Ca_3_Al_2_O_6_, according to a previous protocol.^17^ Comparison of the diffraction pattern of Ca_3_Al_2_O_6_ with that of the literature reference^24^ confirmed its phase purity; see Figure S1 of the Supporting Information, SI. Concerning TCA (Figure S2, SI), it is the major phase, with minor solids being hydroxide and/or carbonate-containing layered double hydroxides (OH- and CO_3_-LDHs), with formulas of [Ca_2_Al­(OH)_6_]_2_(OH)­(CO_3_)·4H_2_O and [Ca_2_Al­(OH)_6_]_2_(CO_3_)·5H_2_O, respectively.^16,17,25^ TCA is prone to transform to such LDHs, especially due to incidental CO_2_ absorption during filtration or long storage. Nevertheless, both the Ca:Al molar ratio and the acid consumption agreed with those expected from the ideal composition of TCA, which will be presented later.

Since Ca_3_Al_2_O_6_ can be readily transformed to TCA,^17^ PKAT phases were prepared first by hydrating Ca_3_Al_2_O_6_ in the presence of SiO_2_ fume (350–420 m^2^ g^–1^, Alfa Aesar) or water glass (a. r. grade, ∼6.5 mol L^–1^ Si, Merck). The solid:liquid ratio was 1:5,^19^ and the nominal degree of substitution, *x*, was varied between 0.1 and 1.0, corresponding to Ca_3_Al_2_(SiO_4_)_0.1_(OH)_11.6_ – Ca_3_Al_2_SiO_4_(OH)_8_. The suspensions were stirred for 2 days at 95 °C; such high temperature was found to facilitate the formation of katoites.^19,20^ However, a mixture of solid phases was obtained, including calcium silicate hydrate (C–S–H),^26^ Al­(OH)_3_,^17^ and sodalite.^27^ (See Figures S3 and S4 and brief discussion in the SI.) Indeed, C–S–H is a common byproduct during katoite synthesis,^19^ whereas sodalites are formed during the predesilication step in the Bayer process.^1,2,5,9,15^

As the hydration method did not yield a phase-pure product, substitution reactions were performed by mixing TCA with water glass (solid:liquid ratio = 1:5) at room temperature by varying *x* from 0 to 1 (solid:liquid ratio = 1:5, *x* = 0–1, *t*_reaction_ = 1 day), or by varying *t*_reaction_ from 1 day to 3 weeks (*x* = 1). Further, as C–S–H and sodalite are possible byproducts (either crystalline or amorphous), reference materials were prepared. Hydroxysodalite (HXS), representing all Bayer sodalites, was made by digesting eckalite kaolin^27^ in a ∼19 mol L^–1^ NaOH solution, prepared from pellets (a.r. grade, VWR Chemicals). C–S–H was made by reacting CaO with water glass with a Ca:Si molar ratio of 1:0.4. Its diffraction pattern showed Ca­(OH)_2_ as a major phase, and polycrystalline C–S–H as a minor phase.^24,26^ The diffractograms of HXS and C–S–H are shown in Figures S5 and S6, SI, whereas their chemical formulas deduced from ICP-MS and TG methods are listed in Table 1.

**Table 1:** Loss on Ignition (LOI), Net Formula, and Unit Cell Constant of Tricalcium Aluminate Hexahydrate (TCA), Pseudokatoite (PKAT), Hydroxysodalite (HXS), and Calcium Silicate Hydrate (C–S–H) Phase Prepared in This Work

solid	formula	LOI/%	cell constant/Å
TCA	Ca_2.9_Al_2_(OH)_11.8_	28.7	12.56–12.58 ^19–21^
PKAT	Ca_3_Al_2_(SiO_4_)_0.9_(OH)_8.4_·3.0H_2_O^a^,^b^Ca_3.08_Al_2_(SiO_4_)_0.05_(OH)_11.96_ ^c^	28.7	12.57
HXS	Na_8_[AlSiO_4_]_6_(OH)_1.4_(CO_3_)_0.3_·2.8H_2_O^d^	7.3	n.d.
C–S–H	Ca(OH)_2_·0.2CaSiO_3_·0.5H_2_O	25.2	n.d.

a Determined from LOI and ICP-MS, assuming that all Si is incorporated into PKAT.

b Sodium was also detected. The Na:Al molar ratio was ca. 0.3.

c Based on the modeled composition (Ca_2.86_Al_1.86_Si_0.043_O_11.74_H_12.00_), assuming the atomic number of Al to be 2.00, and satisfying charge neutrality.

d Carbonate content was calculated based on total carbon determination.

All syntheses were performed in tight-screw borosilicate vessels and a N_2_ atmosphere to minimize carbonation. In all cases, deionized water was used (Milli-Q, Merck Millipore). The solids were filtered using 0.45 μm PTFE/PES membrane filters, washed several times with deionized water, and dried under an infrared lamp in a N_2_ atmosphere.

### Structural Characterization Methods

Powder X-ray diffraction (XRD) patterns of the as-prepared solids and neutralization products were recorded on a Rigaku Miniflex II instrument (with scintillation detector operating at 30 kV and 15 mA) in the range of 2θ = 4–100° with 4° min^–1^ scanning rate, using a Co Kα (λ = 1.7902 Å) radiation source without a monochromator. 2θ­(Co) values were converted to 2θ­(Cu) using the Bragg equation. In the case of Rietveld refinement, the diffractograms were taken by a Philips PW 1830 diffractometer in the range of 2θ = 3–80° with 0.6° min^–1^ scanning rate and 0.02° step width, using Cu Kα radiation at 40 kV and 30 mA. To assess the accuracy of the instrument, the diffractogram of potassium iodide (a.r. grade, VWR Chemicals) was taken, and its characteristic diffraction peaks were compared with reference data (PDF #04–0471);^24^ the differences were smaller than 0.5%.

The structures of TCA and PKAT were probed by a JASCO 4700 FT-IR spectrometer in the range of 500–4000 cm^–1^, applying an optical resolution of 4 cm^–1^, and 128 scans for each spectrum. The spectra were recorded in attenuated total reflection mode, employing a ZnSe crystal and a deuterated triglycine sulfate detector. In addition, TCA and PKAT were compared with the aid of magic-angle-spinning (MAS) for ^27^Al nuclei or ^1^H cross-polarization (CP) MAS nuclear magnetic resonance (NMR) spectroscopy for ^29^Si nuclei. ^27^Al and ^29^Si NMR spectra (128 and 512 scans, respectively) were recorded by means of a Bruker Avance II 400 spectrometer, with a proton frequency of 400.13 MHz (9.38 T). The applied rotation speed was 8000 Hz for ^27^Al and 5000 Hz for ^29^Si, applying 4 mm zirconia (ZrO_2_) rotors with short Vespel caps.

The morphology of PKAT was characterized by scanning electron microscopy (SEM, Hitachi S–4700 II) at various magnifications and acceleration voltages. A few nanometers of conductive gold–palladium alloy film was sublimed onto the surface of the samples to avoid charging. The elemental composition for selected samples was determined via a coupled Röntec QX2 energy-dispersive X-ray spectrometer equipped with a Be window.

The thermal losses of TCA and PKAT were quantified in a TA Instruments Discovery TGA analyzer, operating under air at a 10 °C min^–1^ heating rate. During measurements, 25–30 mg of the sample was calcined in platinum crucibles. The carbon content of as-prepared hydroxysodalite (HXS) was measured with an Analytik Jena N/C 3100 apparatus equipped with an NDIR detector at a furnace temperature of 950 °C.

### Quantitative Analysis

To determine the elemental composition of TCA and PKAT, 10 mg solid was dissolved in a 5 wt % HNO_3_ solution (ICP-MS grade, VWR chemicals). The total concentrations, [X]_T_, of Na, Al, Si, and Ca in these solutions or in supernatants of neutralization experiments were obtained via ICP-MS (model Agilent 7900). All samples were appropriately diluted, and concentrations of Na, Al, Si, and Ca were quantified based on an 11-point calibration series, prepared from a. r. grade salts (NaCl, AlCl_3_·6H_2_O, CaCl_2_·2H_2_O; VWR Chemicals) or from commercial stock solution in the case of Si (1000 mg L^–1^, VWR Chemicals). Each sample contained 1–5 wt % HNO_3_ (ICP-MS grade, VWR Chemicals), as well as 0.1 mg L^–1^ Y and Sc as internal standards (using 1000 m g L^–1^ stock solution, VWR Chemicals).

As for supernatants, the concentration of Si was usually too low for accurate determination by ICP-MS; hence, the blue silicomolybdate method^28^ was employed, allowing for quantification of silicon even for *c*_Si_ < 0.1 mg L^–1^. Samples were prepared according to the literature procedure using a cc. HCl (a. r. grade, 37 wt %, VWR Chemicals), cc. H_2_SO_4_ (a. r. grade, 95–98 wt %, Merck), Na_2_SO_3_ (98%, Thermo Scientific), ammonium heptamolybdate tetrahydrate (a. r. grade, VWR Chemicals), and 4-methylaminophenol sulfate (99%, Thermo Scientific), respectively. The absorbances were measured in quartz cuvettes (Hellma) with a Specord 210 double-beam spectrophotometer (Analytik Jena).

Rietveld analysis of as-prepared PKAT was performed using the HighScore Plus software,^29^ based on reference patterns of katoite (ICDD #00–038–0368), calcite (ICDD #00–005–0586),^30^ and alpha brass (Cu_0.7_Zn_0.3_); the CIF file of the latter was generated with the aid of Gemini.^31^ No alignment calibration was performed prior to refinement. The diffractogram was fitted by using a Pseudo-Voigt function in Highscore Plus. The specimen displacement, scale factor, unit cell dimension, and Caglioti W parameter were refined. Preferred orientation of the specimen was minimal; hence, no correction was applied. The atomic coordinates of the oxygens were refined, and all other positions remained fixed to original values as per symmetry rules. The isotropic displacement factors *B* (Å^2^) for O, Si, Ca, and Al were refined from original values but constrained to 2.0 Å^2^; all values remaining <1.0 Å^2^ after fitting. The site occupancy factors for O, Si, Al, and Ca were also refined.

### Batch Measurements and Potentiometric Titrations

To obtain information on the kinetics of neutralization, TCA or PKAT suspensions were prepared with *c*_TCA_ = *c*_PKAT_ = 10 g L^–1^ (*V* = 20 mL) in polypropylene vials, using deionized water. 2.0 equiv of 1.005 mol L^–1^ HCl was added, where the acid equivalent reads as

$$\mathrm{acid}\;\mathrm{equivalent}=\frac{n_{\mathrm{HCl}}}{n_{\mathrm{solid}}}$$ 5

Here, *n*_solid_ was calculated from the molar mass of the nominal composition obtained from ICP-MS and TG (Table 1). The suspensions were stirred at (23 ± 2)°C up to 7 days, and the pH readings were carried out regularly, using a SenTix H glass electrode (Xylem Analytics), calibrated against commercial buffer solutions (VWR Chemicals) in the pH range of 1.68–13.0. For PKAT, another set of samples with 3 equiv of HCl was prepared; the samples were stirred and filtered after different reaction times (up to 2 days), using 0.45 μm PTFE/PES membrane filters, washed with deionized water, and dried under N_2_ atmosphere.

Acid-dependent batch experiments were performed by preparing sample series for both solids with *c*_TCA_ = *c*_PKAT_ = 10 g L^–1^ (*V* = 20 mL) with varying concentrations of HCl from 0 to 6.0 equiv. In each case, the pH was measured, and samples were filtered after 2 days; the respective solid and liquid phases were then analyzed.

Furthermore, acid-dependent titration experiments carried out with an 888 Titrando automatic titrator (Metrohm). Here, TCA or PKAT suspensions (*c*_TCA_ = *c*_KAT_ = 10 g L^–1^, *V* = 40 mL) were titrated with 1.005 mol L^–1^ HCl at (25.0 ± 0.1)°C in a PTFE vessel, applying N_2_ atmosphere and stirring. The total added volume of HCl was 12.4–13.2, corresponding to acid equivalents of >11.5 (TCA) and >12.3 (PKAT), respectively. 1 or 2 h of contact times were set before the first or between subsequent aliquots; the 2 h experiments were made in triplicate for PKAT. The pH was monitored with a calibrated glass electrode (SenTix H).

## Results and Discussion

### Syntheses and Characterization of TCA and PKAT

Reacting TCA with water glass at room temperature, we find that irrespective of *x*, the patterns of PKAT are essentially identical to those of the precursor TCA (Figure S7, SI), except that LDH phases are absent. Consequently, lower synthesis temperature is favorable concerning the phase stability of TCA, whereas elevated temperature induces the formation of silicate-containing byproducts. Given the close similarity between as-prepared katoites and TCA, we will use the term “pseudokatoite” (PKAT) to distinguish them from high silica-substituted, “real” katoites.

For PKAT with *x* = 1, i.e., Ca_3_Al_2_SiO_4_(OH)_8_, increasing the reaction time from 1 day to 3 weeks yielded solids which are virtually free of any crystalline byproduct (Figure 1a). The phases obtained after 2 days or 1 week appeared to be the least amorphous. Considering that longer mixing time allows for more homogeneous elemental distribution in the forming phase, PKAT with a 1 week reaction time was chosen for further experiments.

**Figure 1:**
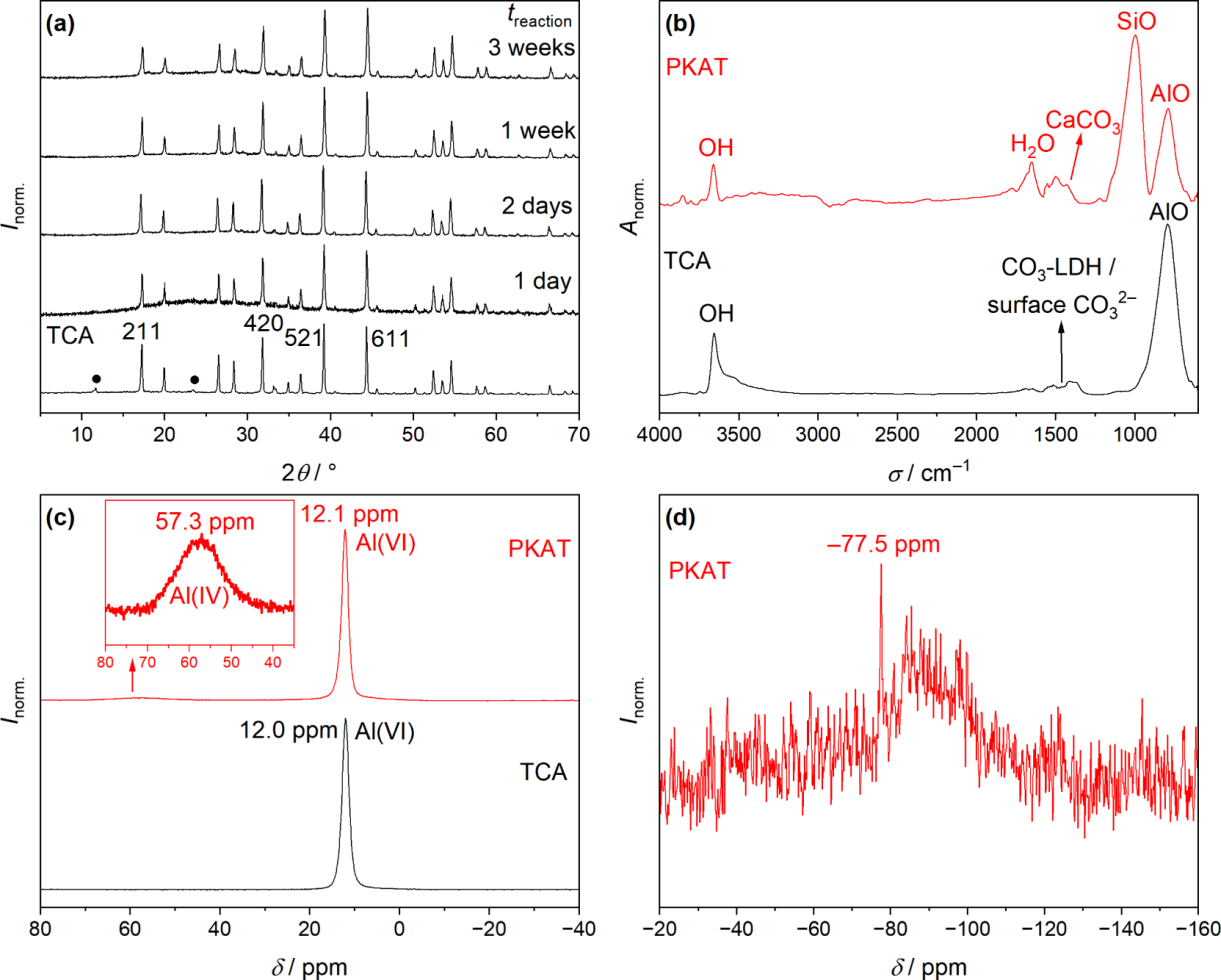
(a) Powder X-ray diffractograms of pseudokatoites (PKATs), Ca_3_Al_2_(SiO_4_)*_*x*_* (OH)_12–4*x*_, prepared by reacting Ca_3_Al_2_(OH)_12_ (TCA) with Na_2_SiO_3_ solution applying different reaction times (room temperature). The degree of substitution *x* = 1 is the target value. As reference, TCA and the Miller indices of its four most intense diffractions^24^ are shown. Black symbols represent OH/CO_3_ layered double hydroxide phases. (b) Infrared spectrum of Ca_3_Al_2_(SiO_4_)_0.9_(OH)_8.4_ (PKAT), corresponding to *t*_reaction_ = 1 week in panel (a), and that of TCA. (c) ^27^Al MAS NMR spectrum of PKAT and TCA. The inset shows the peak belonging to tetrahedral Al. (d) ^29^Si CP-MAS NMR spectrum of PKAT, where the peak at −77.5 ppm indicates Q^0^ or Q^1^-type Si nuclei. In each panel, data are normalized such that the highest value is unity.

Contrary to XRD, the IR spectra of PKAT and TCA are markedly different (Figure 1b). The dominant vibration band of TCA is located at 790 cm^–1^ and can be assigned to the Al–O stretching.^23^ The same band is present also for PKAT, but a more intense one appears at 1000 cm^–1^, which stems from Si–O stretching with Q^1^- and Q^2^-type bonding environments, associated with either Al or Ca atoms.^23,32–36^ Further, the signal of calcite (∼1420 cm^–1^, PKAT) and asymmetric/symmetric vibrations are observed in the range of 1300–1600 cm^–1^, arising from either surface carbonate with monodentate coordination^34,37^ or interlayer carbonate of CO_3_-LDH phases (TCA).^38^ The band at 1650 cm^–1^ corresponds to the bending vibration of water molecules,^32,35–37^ which is much more pronounced for PKAT than for TCA. This is supported by the TG curves (Figure S8, SI), which show significantly more surface or hydration water for PKAT than for TCA, indicating hydrous by-products in the former solid. Yet, the total mass losses, dominated by dehydroxylation at 300–320 °C, which are typical for katoites,^17,33^ are the same for both phases (Table 2). Finally, the sharp peak at 3650 cm^–1^ for both phases stems from the stretching vibration of individual OH^–^ ions.^18,32^

The ^27^Al NMR spectra of the two solids (Figure 1c) exhibit a peak at ∼12 ppm, which can be attributed to the resonance of ^27^Al nuclei in an octahedral environment.^39–41^ This chemical shift matches the peak position (12.4 ± 0.5 ppm) reported earlier for TCA.^40^ Interestingly, a second, low-intensity peak is seen for PKAT at ∼57 ppm (Figure 1c, inset), arising from tetrahedral nuclei.^39^ In this respect, Al-bearing C–S–H phases (C–A–S–H) hosting tetracoordinated aluminum atoms showing up at >60 ppm.^33,41,42^ Consequently, the peak at 57 ppm might stem from a C–A–S–H amorphous phase. Besides, it may be assigned to the presence of amorphous sodium aluminosilicate: indeed, peaks at 52 and 60 ppm have been reported for sodium aluminosilicate gel and Y zeolite, respectively.^43,44^ The presence of Na^+^ in PKAT, based on ICP-MS measurements (Table 1), supports this assumption.

The ^29^Si spectrum of PKAT shows a hump between −75 and −115 ppm (Figure 1d), indicating an amorphous C–S–H phase with broad distribution of Q^1^ and Q^2^ Si environments.^23,33,39,42,45^ There is, however, a sharp resonance peak at −77.5 ppm, which could be assigned to Q^0^ Si linked only to Al­(OH)_6_ octahedra in katoites (−80 ppm),^40^ or to Q^1^ Si in C–S–H/C–A–S–H phases (−80 to −76 ppm).^32,33,45^ Finally, the scanning electron micrograph shows mainly micrometer-sized aggregates of irregular shape (Figure S9a, SI), similar to TCA,^17^ and to katoites prepared with SiO_2_ at 60 °C, too.^46^ In addition, the elemental maps show a homogeneous distribution for Al, Si, and Ca (Figure S9c,d, SI).

### Composition of TCA and PKAT

Elemental analysis shows the Ca:Al molar ratio to be very similar to the ideal one (3:2) for both solids; see Table 1. For PKAT, *x* ≈ 0.9, which yields a nominal formula of Ca_3_Al_2_(SiO_4_)_0.9_H_8.4_. Since the target value of *x* was 1, this means that most of the initial amount of silica has been incorporated into TCA. Conversely, qualitative X-ray diffractograms indicate negligible structural difference between the two phases (Figure 1a), suggesting *x* to be ≪0.9.

This apparent contradiction can be resolved by quantitative structure analysis via Rietveld refinement (Figure 2). Indeed, as-prepared cubic PKAT has the same cell constant (12.57 Å) as that of TCA, 12.56–12.58 Å;^19–21^ see fitted parameters in Table 2. Further, the calculated composition of PKAT is indeed very close to that of TCA, with *x* being ∼0.05, corresponding to Ca_3.08_Al_2_(SiO_4_)_0.05_(OH)_11.96_. We obtained this formula based on the modeled composition in Table 2, assuming the stoichiometric number of Al to be 2.00 and obeying electroneutrality. In conclusion, most of the Si counted by ICP formed an amorphous (thus nondiffracting) silicate phase instead of replacing OH^–^ ions of TCA, in line with the observation that formation of katoite is kinetically hindered at ambient temperature.^20^ In addition to poorly substituted TCA, PKAT also contains 3.2 wt % calcite (in line with the infrared spectrum in Figure 1b), which formed via surface carbonation. Note that PKAT was free of calcite right after synthesis, as diffractograms in Figure 1a and Figure S7, SI do not show calcite diffraction peaks. However, quantitative XRD could not be measured immediately, and CaCO_3_ formed during sample storage had to be taken into account for an acceptable fit.

**Figure 2:**
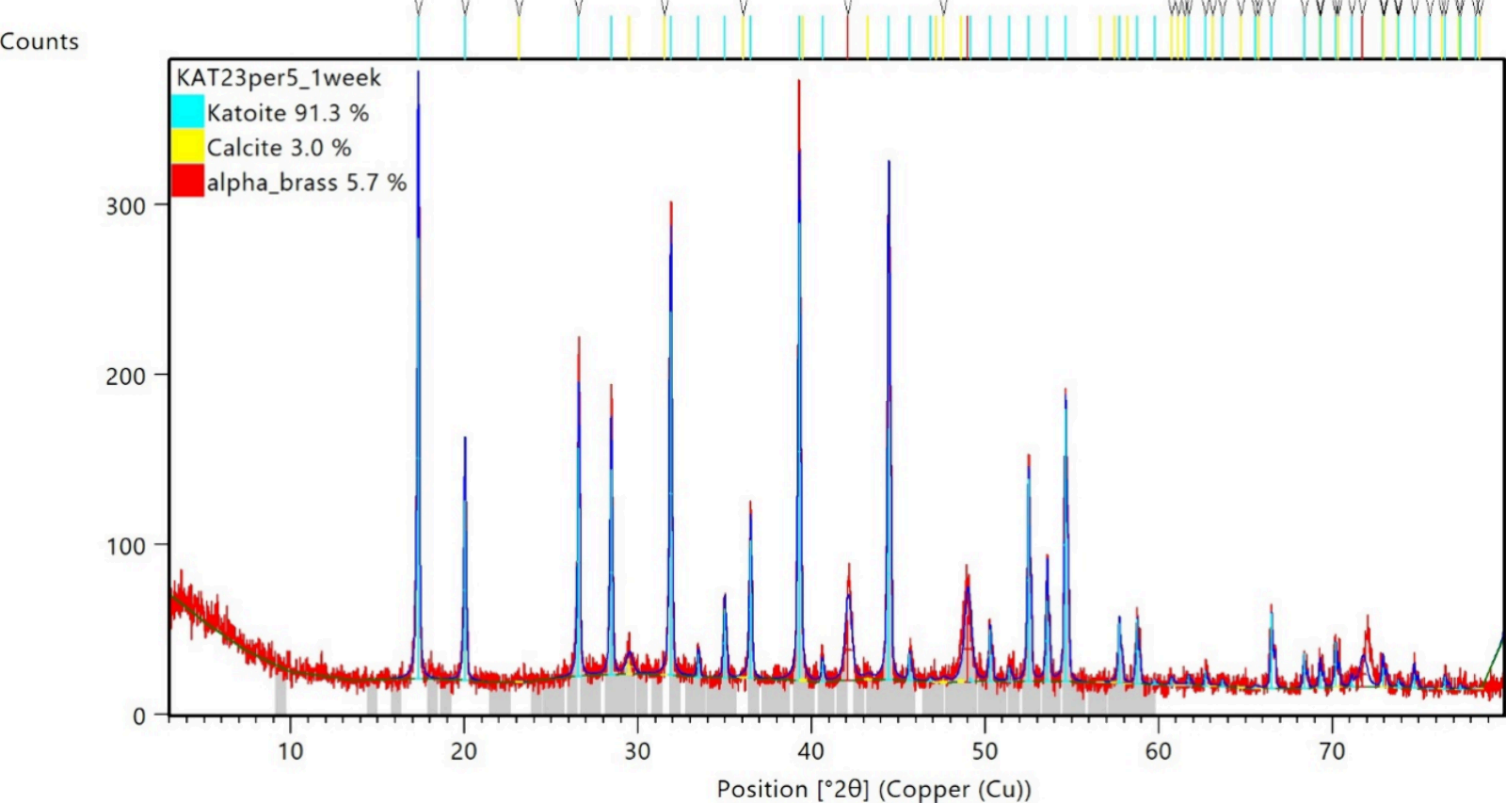
Rietveld refinement of pseudokatoite (PKAT), Ca_3_Al_2_(SiO_4_)_0.9_(OH)_8.4_ (nominal composition obtained by ICP-MS). In addition to katoite and calcite, diffractions of alpha brass were discernible, originating from the sample holder. Subtracting its contribution, the phase composition is 96.8 wt % PKAT and 3.2 wt % calcite.

**Table 2:** Fitted Parameters for PKAT (Ca_3_Al_2_(SiO_4_)_0.9_(OH)_8.4_) as Obtained by Rietveld Refinement, Based on Reference Patterns #00–038–0368 and #00–005–0586 of the International Centre of Diffraction Data^30^ ^a^

**atom**	model	UCF	S.O.F.	charge	*a* = *b* = *c*/Å	*V*/Å^3^	*R*_*wp*_
H	96.00	12.00	1.00	12.00	12.572	1987.2	17.7
O	93.94	11.74	0.98	–23.49			
Si	0.34	0.043	0.04	0.17			
Al	14.90	1.86	0.93	5.59			
Ca	22.89	2.86	0.95	5.72			

a The thus calculated phase composition is Ca_2.86_Al_1.86_Si_0.043_O_11.74_H_12.00_.

In summary, we find that PKAT is virtually identical to TCA; however, it most likely contains amorphous C–S–H and sodium aluminosilicate byproducts. These minor phases give rise to dramatically different neutralization behavior as compared to TCA, which is discussed in the following sections.

### Neutralization of TCA and PKAT

#### Time-Dependent Batch Experiments

We first studied the time-dependence of the neutralization of the two solids, respectively, by adding 2 equiv of HCl to their 10 g L^–1^ suspension (Figure 3a,b). For both solids, the system reaches steady-state pH in 2 days. This constant pH is markedly higher for TCA than for PKAT by ca. 0.8 pH units (∼11.5 vs ∼10.8), signaling a lower buffering pH range of the latter. Moreover, we see a similar trend for batch sample measurements for PKAT at 3 equiv acid. (Batch means that a separate sample has been prepared for each reaction time.) These data indicate again that a steady-state pH of ∼10.5 is attained in 2 days, which is however smaller than ∼10.8 due to the larger amount of HCl (3 vs 2 equiv).

**Figure 3:**
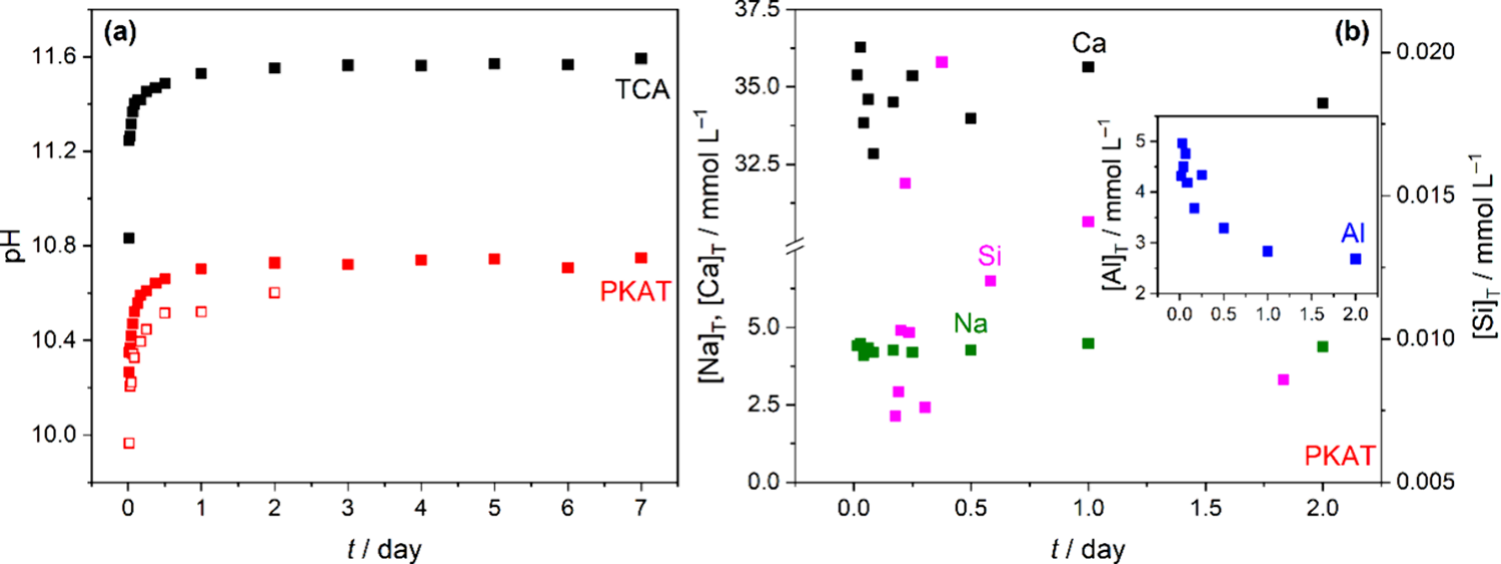
(a) pH evolution over time of Ca_3_Al_2_(OH)_12_ (TCA) and Ca_3_Al_2_(SiO_4_)_0.9_(OH)_8.4_ (PKAT, nominal composition) of 10 g L^–1^ suspensions, following the addition of 2 (full symbols) or 3 (empty symbols) equiv HCl. In the case of 2 equiv of HCl, pH was monitored continuously in one sample, whereas individual samples were prepared for each time point in the case of 3 equiv of HCl. (b) Total molar concentrations, [X]_T_, of Na and Ca (left axis), Si (right axis), and Al (inset), of samples with 3 equiv of HCl in panel (a). The estimated error for pH and [X]_T_ is ± 0.15 and ± 5%, respectively.

The elemental concentrations in the supernatants of the same PKAT samples are shown in Figure 3b. Accordingly, the most soluble component is Ca^2+^, but its concentration appears to reach a maximum (∼35 mmol L^–1^) at early times, indicating fast katoite dissolution. Interestingly, Na^+^ is also detectable, similarly to the initial as-prepared PKAT, and its concentration scatters around 4.3 mmol L^–1^. This suggests that a sodium-containing solid phase dissolves upon reaction with acid, hinting at the presence of sodium aluminosilicate. Note that if the 4.3 mmol L^–1^ Na^+^ had entirely originated from residual NaOH (due to insufficient washing), the respective pH would have been ∼11.6, much higher than the observed value of ∼10.5.

Concerning [Si]_T_, it is smaller than [Ca]_T_ by more than 3 orders of magnitude (0.007–0.02 mmol L^–1^), as it is most likely to be controlled by sparingly soluble calcium silicates. (For pure CaH_2_SiO_4_, an equilibration silicate concentration of 0.3 mmol L^–1^ can be calculated at ideal dilution, based on its log *K*_sp_.^47^) Nevertheless, it shows a maximum after 6 h, possibly due to the initial dissolution of PKAT or amorphous sodium aluminosilicate, followed by the reprecipitation of C–S–H induced by the high [Ca]_T_ in solution.

[Al]_T_ (Figure 3b, inset) exhibits a gradual decrease over time, indicating the following precipitation reaction:

$$\mathrm{Al}{{\left(\mathrm{OH}\right)}_{4}}^{-}\left(\mathrm{aq}\right)⇌\mathrm{Al}{\left(\mathrm{OH}\right)}_{3}\left(s\right)+{\mathrm{OH}}^{-}\left(\mathrm{aq}\right)$$ 6

In addition, Al­(OH)_3_ precipitation yields Ca:Al molar ratios of 8–13, much higher than 3:2 found in the as-prepared PKAT (Table 1),. Equation 6Equation 6 also explains (at least partially) the increase in pH over time (Figure 3a) for both PKAT and TCA, where aluminate ions are always the dominant Al-bearing solution species in alkaline medium.^17,20^

The above notion is supported by changes in the solid phase over the course of the neutralization period (Figure 4). The reference diffractogram of as-prepared PKAT, stirred in water for 2 days, shows the appearance of gibbsite, Al­(OH)_3_ at ∼18.2° and that of calcite (or C–S–H) at ∼29.5°.^26,30,48^ Upon addition of 3 equiv of HCl, we again find the emergence of Al­(OH)_3_ over time, which becomes a major crystalline phase 2 days after the addition of HCl (pH = 10.6). Note that apart from Al­(OH)_3_, CaCO_3_ is present as a minor phase in each solid, originating from the surface carbonation of as-prepared PKAT, due to long storage.

**Figure 4:**
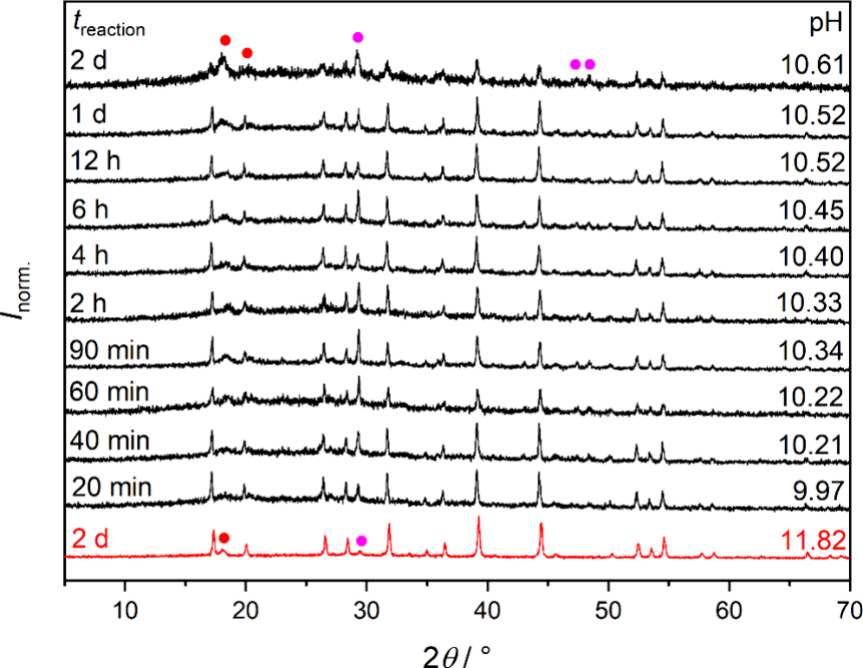
Powder X-ray diffractograms of Ca_3_Al_2_(SiO_4_)_0.9_(OH)_8.4_ (PKAT, nominal composition) as a function of neutralization reaction time, following the addition of 3 equiv of HCl. As reference, PKAT stirred in water for 2 days is also shown (red pattern). In addition to PKAT, other solid phases are represented by symbols; red: gibbsite, magenta: calcite. Diffraction intensities are normalized such that the highest value is unity. Further, pH values correspond to the respective solution phases measured before filtration, and they are identical to those in Figure 3a.

Overall, the dissolution of PKAT is rapid, as indicated by [Ca]_T_ (Figure 3b), while [Al]_T_ declines over time owing to Al­(OH)_3_ precipitation, giving rise to an increase in pH. (Ca^2+^ ions arising from CaCO_3_ are negligible due to its very low solubility.^49^) Most surprisingly, acidification of TCA leads to the formation of Friedel’s salt or Cl-LDH ([Ca_2_Al­(OH)_6_]_2_Cl_2_·4H_2_O) within the first hour,^17^ while this phase is absent for PKAT. Since Cl-LDH has a similar solubility as that of TCA at ideal dilution,^12^ transformation of TCA to LDH maintains high pH, hence a higher buffering range for TCA as compared to PKAT (Figure 3a).

#### Acid-Dependent Batch Experiments

Next, we studied the neutralization behavior of PKAT and TCA as a function of the added amount of acid, ranging between 0 and 6 equiv. The pH values of saturated solutions of TCA and PKAT are 12.0 and 11.8, respectively, suggesting that the dissolving portion of the solids in terms of equilibrium OH^–^ concentrations is similar.

Adding HCl, steady-state pH values for PKAT obtained after 2 days are consistently lower by 0.5–1.2 units than those for TCA samples (Figure 5a), in line with the time-dependent experiments (Figure 3a). Near-neutral pH is attained for TCA at the expected 6 equiv of added acid (eq 3), whereas the pH of PKAT decreases steeply to ∼8 already at 5 equiv HCl. Consequently, the apparent acid consumption of PKAT is smaller by ∼17% as compared to TCA.

**Figure 5:**
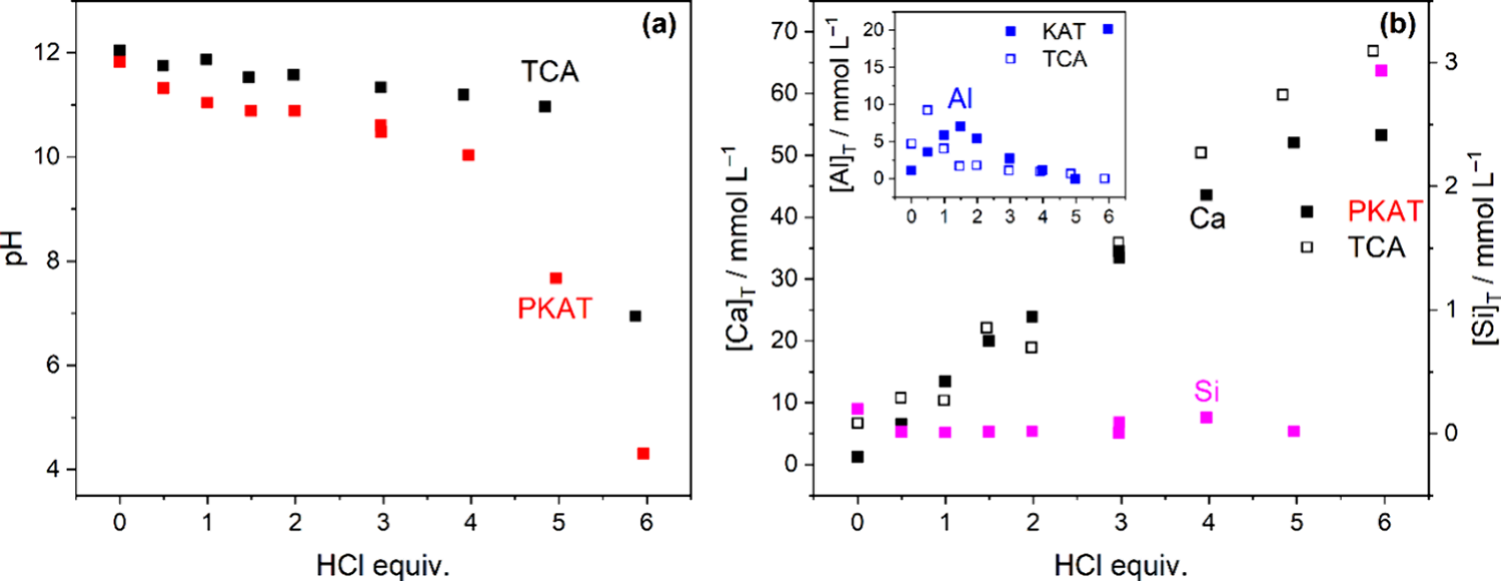
(a) Dependence of pH on added HCl equivalent for Ca_3_Al_2_(OH)_12_ (TCA) and Ca_3_Al_2_(SiO_4_)_0.9_(OH)_8.4_ (PKAT, nominal composition) of 10 g L^–1^ suspensions; *t*_reaction_ = 2 days. (b) Total molar concentrations, [X]_T_, of Ca (left axis), Si (right axis), and Al (inset). Full symbols correspond to PKAT, and empty symbols represent TCA. The pH values in panel (a) and concentrations in panel (b) were measured in the same solutions. The estimated error for pH and [X]_T_ is ± 0.15 units and ± 5%, respectively.

The total concentrations of Al, Si, and Ca corresponding to these pH values are plotted in Figure 5b. Mixing the two solids with water for 2 days yields [Ca]_T_ and [Al]_T_ = 1.1 and 1.2 mmol L^–1^ for PKAT, as well as 6.7 and 4.7 mmol L^–1^ for TCA. For the latter, the Ca:Al molar ratio is 1.4 in solution, which is very close to the ideal 1.5 in the solid phase, evidencing its congruent (regular) dissolution.^17,20^ For PKAT, the ratio is 1.1, suggesting that its dissolution is incongruent (irregular), which has been reported for katoites with higher degrees of substitution as well.^19,20^ Note that irregular dissolution is associated with concurrent dissolution of multiple phases (e.g., PKAT + C–S–H),^19^ or with phase transformation accompanying dissolution of a pure phase (e.g., PKAT → Al­(OH)_3_; Figure 4).

For both TCA and PKAT, the change in [Al]_T_ displays a maximum at 0.5 equiv (TCA) or 1.5 equiv HCl (PKAT). For TCA, [Al]_T_ increases due to the transformation of TCA to Cl-LDH, yielding Al­(OH)_4_^–^ ion (eq 2).^17^ Indeed, we observe the complete transformation of TCA to Cl-LDH already at 1 equiv added HCl, as shown by the diffractograms in Figure 6a. For PKAT, LDH formation is absent (Figure 6b); thus, the increase in [Al]_T_ is related to its simple dissolution (and to a smaller degree to the dissolution of amorphous aluminosilicate):

$${\mathrm{Ca}}_{3}{\mathrm{Al}}_{2}{\left({\mathrm{SiO}}_{4}\right)}_{x}{\left(\mathrm{OH}\right)}_{12-4x}+\left(4-x\right)H^{+}⇌3{\mathrm{Ca}}^{2+}+2\mathrm{Al}{{\left(\mathrm{OH}\right)}_{4}}^{-}+{{xH}_{3}{\mathrm{SiO}}_{4}}^{-}+\left(4-4x\right)H_{2}O$$ 7

**Figure 6:**
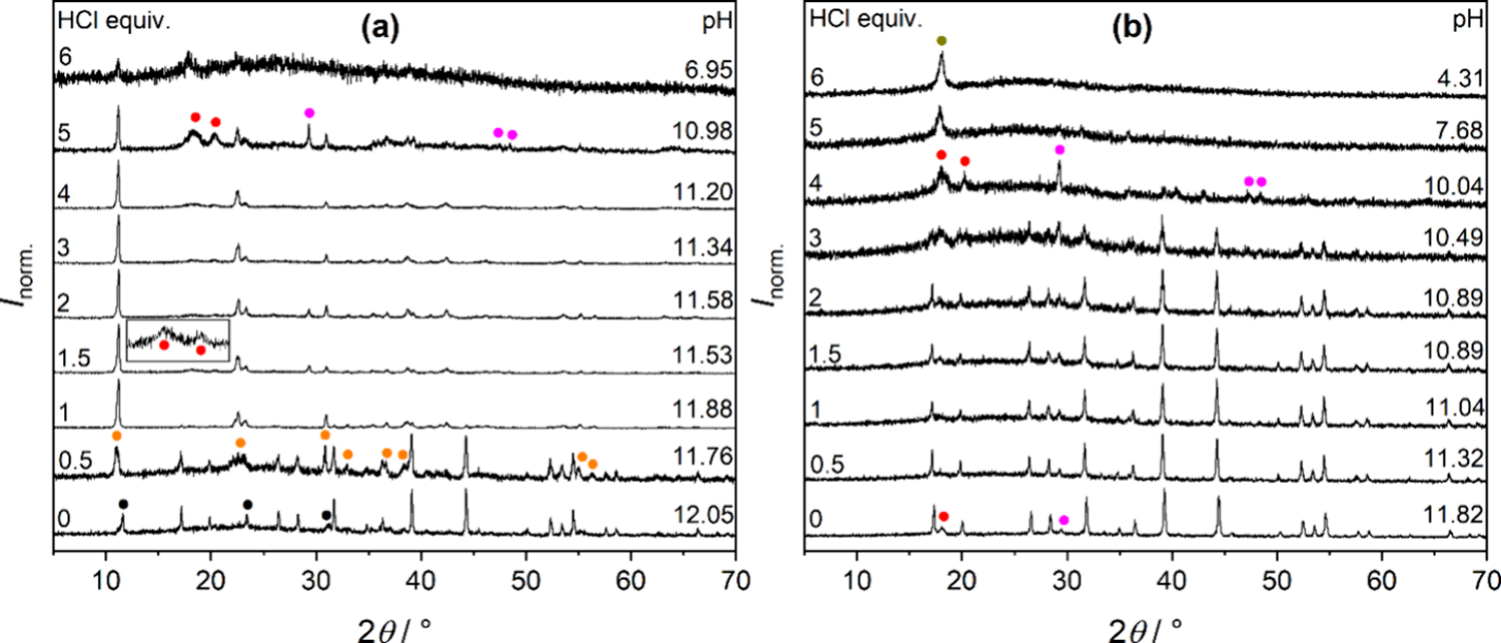
Powder X-ray diffractograms of (a) Ca_3_Al_2_(OH)_16_ (TCA) and (b) Ca_3_Al_2_(SiO_4_)_0.9_(OH)_8.4_ (PKAT, nominal composition) as a function of added HCl equivalent (*t*_reaction_ = 2 days). In addition to TCA and PKAT, peaks of other solid phases are marked by symbols; black: CO_3_-LDH, orange: Cl-LDH, red: gibbsite; magenta: calcite; dark yellow: PTFE.^50^ (PTFE particles formed due to intense mixing of suspensions using PTFE stirring bars.) The inset in panel (a) shows the zoomed region of 12–18° at 1.5 equiv HCl. Diffraction intensities are normalized such that the highest value is unity. Further, pH values correspond to the respective solution phases measured before filtration and they are identical to those in Figure 5a.

Given the two deprotonation constants of H_4_SiO_4_, p*K*_1_ = 11.8 and p*K*_2_ = 9.8,^47^ the predominant form of Si in solution is H_3_SiO_4_^–^ up to 4 equiv of added HCl. Expected from eq 7, [Si]_T_ should increase parallel to [Al]_T_, which is not the case (Figure 5b), as it remains constant in the entire alkaline pH regime. This suggests that dissolving Si­(IV) reprecipitates as amorphous calcium silicate or calcium silicate hydrate.

Further addition of HCl gives rise to a decline in [Al]_T_ owing to gibbsite precipitation for both TCA and PKAT (eq 6), evidenced by the appearance of Al­(OH)_3_ at 18.2° and 20.3° (Figure 6a,b). Concerning [Ca]_T_, it gradually increases up to 6 equiv HCl for TCA, while it plateaus at 5 equiv HCl for PKAT. This difference agrees with the lower acid consumption of PKAT (Figure 5a), suggesting that a significant fraction of this solid is nondissolving down to pH ≈ 8.

Addition of 6 equiv of HCl to PKAT yields an acidic pH, giving rise to a drastic increase in both [Al]_T_ and [Si]_T_, indicating enhanced dissolution of gibbsite and Si-containing phases. It is worth mentioning that CaCO_3_ is also present as a minor phase, but its dissolution is negligible due to its low solubility in alkaline media.^49^ As such, it only dissolves below pH ≈ 8 (Figure 6b), where it contributes to [Ca]_T_ determined by its fraction in PKAT (3.2 wt %) as obtained from Rietveld analysis.

In summary, PKAT dissolves without the Cl-LDH intermediate, indicating that silica (in certain or all forms) might act as “crystal poison” for Friedel’s salt, i.e., it hinders the precipitation of the LDH phase. Supporting this observation, little to no Cl-LDH formed when silica fume was added to chloride-containing concrete samples.^51^ Further, the tendency of solids to form LDH decreased in the order of hydrated tricalcium aluminate + gypsum > hydrated Portland cement > hydrated tricalcium silicate, with no LDH in the third case ([Cl]_T_ ranged between 0.001 and 5 mol L^–1^).^52^ However, Cl-LDH formation was evident for alumina-blended clinker at [Cl]_T_ = 0.5 mol L^–1^ or hardened cement paste at [Cl]_T_ ≥ 1 mol L^–1^.^53,54^ These results highlight the critical role of the type of silicate phase (and possibly the total chloride concentration) in LDH formation and stability.

#### Titration Measurements

We now turn to the titration curves of TCA and PKAT. TCA (Figure 7a) exhibits minor differences in pH when comparing data obtained by applying either 1 or 2 h as dwell time between two aliquots of added HCl. Further, there is a good agreement between titration and batch data (*t*_reaction_ = 2 days), indicating a fairly rapid equilibration time for TCA. Conversely, pH values above this amount of acid are much lower (<3.1) than those in equilibrium (∼4), indicating slow dissolution of gibbsite, which is the buffering phase in acidic medium.^17^

**Figure 7:**
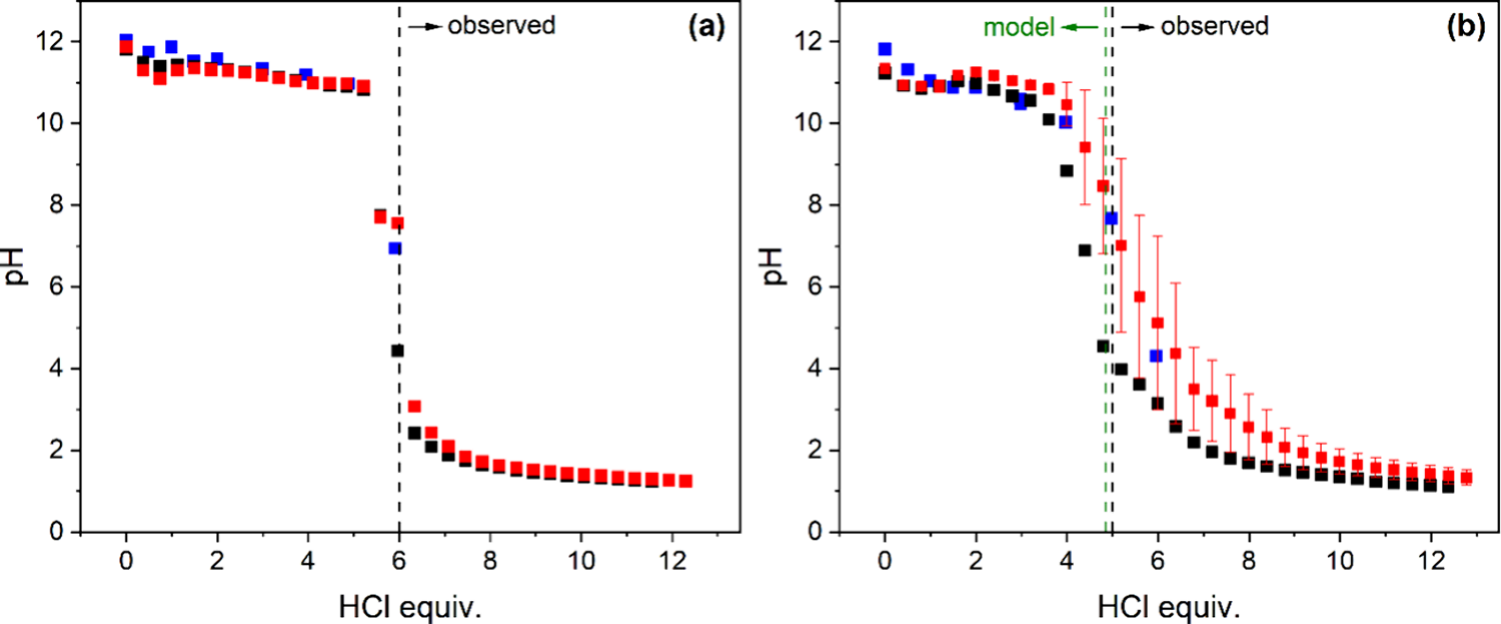
Potentiometric titration curves as a function of added HCl equivalent for (a) Ca_3_Al_2_(OH)_12_ (TCA) and (b) Ca_3_Al_2_(SiO_4_)_0.9_(OH)_8.4_ (PKAT, nominal composition) applying 1 h (black symbols) or 2 h (red symbols) waiting time between each aliquot. The initial concentration of the suspensions was 10 g L^–1^, and that of titrant HCl was 1 mol L^–1^. Also shown are data obtained after 2 days of reaction time for individual samples with the same suspension concentration. The error bars in panel (b) are the standard deviations of three parallel titrations. Black dashed lines in both panels pertain to the observed acid consumption, required to reach pH ≈ 7. The green dashed line in panel (b) is based on the model; see the Section “Modeling the Acid Consumption of PKAT”.

Concerning PKAT (Figure 7b), titration data taken with different equilibration times are in fair agreement with each other in the alkaline range (<4 equiv or pH > 10), although pH values obtained from titrations exhibit a minimum at 1 equiv HCl, indicating that equilibrium has not yet been attained by the system. Upon further neutralization, data with a 1 h equilibration time are significantly smaller than those with 2 h and 2 days, respectively. In addition, the error bars of the 2 h triplicate measurements are large, suggesting very slow dissolution in the weakly alkaline and acidic ranges. Since [Si]_T_ markedly increases from 5 to 6 equiv acid (Figure 5b) if the suspensions are allowed to equilibrate for 2 days, slow kinetics is certainly related to the dissolution of C–S–H phases present in PKAT. In fact, the dissolution of wollastonite, CaSiO_3_, has been reported to be very slow, ranging from 10^–14^ (pH ≈ 12) and 10^–10^ mol m^–2^ s^–1^ (pH ≈ 0).^55^ (It has to be mentioned that, in addition to slower kinetics, possible formation and random deposition of silica gel at various spots of the PTFE titration vessel might have contributed to the observed experimental errors.)

In addition to the kinetic features of the neutralization behavior of the two solids, another striking difference between them is the equivalence point, i.e., the point corresponding to the transition from the alkaline to the acidic range (dashed lines in Figure 7a,b). This is 6 equiv in the case of TCA, in agreement with eq 3. and previous findings.^17^ As for PKAT, both titration and batch data suggest that this point is at ∼5 equiv, which means that this solid consumes ca. 17% less acid than TCA. This apparently contradicts the notion that PKATs with any degree of substitution should also consume 6 equiv HCl:

$${\mathrm{Ca}}_{3}{\mathrm{Al}}_{2}{\left({\mathrm{SiO}}_{4}\right)}_{x}{\left(\mathrm{OH}\right)}_{12-4x}+6H^{+}⇌3{\mathrm{Ca}}^{2+}+2\mathrm{Al}{\left(\mathrm{OH}\right)}_{3}+xH_{4}{\mathrm{SiO}}_{4}+\left(6-4x\right)H_{2}O$$ 8

Equation 8 is based on the fact that dissolving SiO_4_^4–^ ions become fully protonated at neutral pH (similar to OH^–^ ions), based on the deprotonation constants of silicic acid.^47^ A possible interpretation of the smaller acid consumption of PKAT is discussed below.

### Modeling the Acid Consumption of PKAT

Both structural characterization and Rietveld analysis point to the strong similarity between TCA and PKAT. Yet, PKAT shows ∼17% less acid consumption compared to that of TCA. It is possible to account for this difference by considering the two formulas obtained by Rietveld analysis (Ca_3.08_Al_2_(SiO_4_)_0.05_(OH)_11.96_) and ICP-MS (Ca_3_Al_2_(SiO_4_)_0.9_(OH)_8.4_); see Table 1. We hypothesize that the sources of excess silica in the latter are amorphous C–S–H and sodium aluminosilicate, which (together with CaCO_3_) consume much less acid than Ca_3.08_Al_2_(SiO_4_)_0.05_(OH)_11.96_. That is, only a fraction of PKAT (<100 wt %) reacts with 6 equiv HCl, thus requiring less than 6 equiv to reach pH ≈ 7.

To model the acid consumption, we assume that 1 g of PKAT is composed of 0.032 g CaCO_3_ (or 3.2 wt %, Figure 2), *y* g of Ca_3.08_Al_2_(SiO_4_)_0.05_(OH)_11.96_, *z* g of CaH_2_SiO_4_ (as the simplest model of C–S–H phases) and (0.968–*x*–*y*) g of NaAlSiO_4_ (as the simplest model of sodium aluminosilicates). Since molar ratios of Ca:Al = 1.5 and Si:Al = 0.45 are known from ICP-MS (Table 2), equations for these ratios with respective molar masses can be written and solved.

Accordingly, PKAT contains 69.9 wt % Ca_3.08_Al_2_(SiO_4_)_0.05_(OH)_11.96_, 12.9 wt % NaAlSiO_4_, 14.0 wt % CaH_2_SiO_4_, and 3.2 wt % CaCO_3_. The acid consumption of the Ca_3.08_Al_2_(SiO_4_)_0.05_(OH)_11.96_ fraction is 6 equiv (eq 8). Moreover, the dissolution of calcite at 5. equiv is evident from the diffraction pattern (Figure 6b), suggesting the acid consumption to be 1 equiv due to the CO_3_^2–^ → HCO_3_^–^ protonation reaction. NaAlSiO_4_ also has to be considered as an acid-consuming component since time-dependent experiments show the presence of Na^+^ ions in solution. Supporting this, hydroxysodalite would yield [Na]_T_ ≈ 1.5 mmol L^–1^ at 25 °C and infinite dilution,^56^ which is in the same order of magnitude as those measured in this work (∼4.3 mmol L^–1^; Figure 3b). Here, the acid consumption of NaAlSiO_4_ is assumed to be 1 equiv, according to eq 9:

$$\mathrm{NaAl}{\mathrm{SiO}}_{4}+H^{+}+3H_{2}O⇌{\mathrm{Na}}^{+}+\mathrm{Al}{\left(\mathrm{OH}\right)}_{3}+H_{4}{\mathrm{SiO}}_{4}$$ 9

Concerning CaH_2_SiO_4_, its acid consumption can be considered negligible up to 5 equiv HCl due to its low solubility^47^ and the very low values of [Si]_T_ (Figures 3b and 5b).

Consequently, Ca_3.08_Al_2_(SiO_4_)_0.05_(OH)_11.96_ together with NaAlSiO_4_ and CaCO_3_ consume 12.05 mL of HCl with respect to 1 g of PKAT. Based on its net formula (Ca_3_Al_2_(SiO_4_)_0.9_(OH)_8.4_; Table 2), PKAT would require, however, 14.93 mL. Thus, the calculated acid consumption is 12.05/14.93·6 equiv = 4.84 equiv, which is very close to the observed 5 equiv shown by the titration curves and batch pH measurements (Figure 7b).

This model can be further supported by comparing measured and calculated [Ca]_T_ values of batch experiments, as displayed in Figure 8. First, we calculate the expected [Ca]_T_ values, [Ca]_T,exp._, assuming that Ca^2+^ ions are released into the solution phase only if HCl is added. Since the total consumption is 6 equiv and each PKAT unit contains 3 Ca^2+^ ions:

$$[\mathrm{Ca}{]}_{T,\exp.}={\left[\mathrm{HCl}\right]}_{T}\times \frac{3}{6}$$ 10

where [HCl]_T_ is the total concentration of added acid. It is straightforward from eq 6 that [Ca]_T,exp._ depends on the amount of added acid, not on the actual composition of PKAT. The thus obtained [Ca]_T,exp._ data agree very well with the measured ones, [Ca]_T,meas._, indicating that PKAT readily dissolves upon neutralization. Conversely, [Ca]_T,exp._ deviates and becomes smaller than [Ca]_T,meas._ exactly at 5 equiv HCl, indicating that maximum solubility is reached at this point (pH ≈ 7.7).

**Figure 8:**
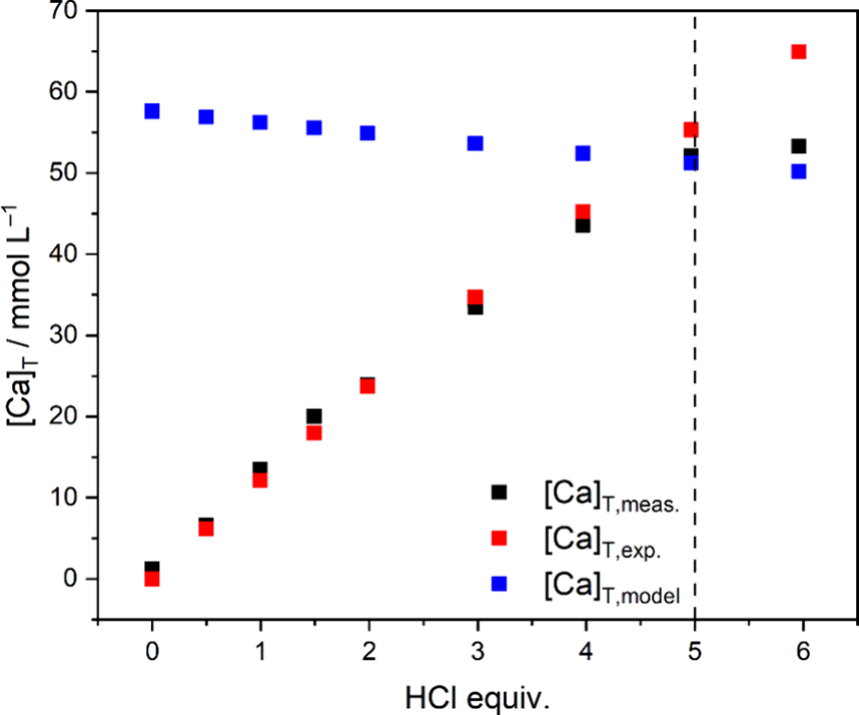
Total molar concentration of Ca, [Ca]_T_, as a function of the added HCl equivalent. Shown are measured data ([Ca]_T,meas._, black symbols), expected concentrations ([Ca]_T,exp._, red symbols) based on eq 10, and modeled values ([Ca]_T,model_, red symbols) based on eq 11. The dashed line at 5 equiv represents the point where [Ca]_T,model_ and [Ca]_Tmeas_ coincide. The estimated error for [Ca]_T,meas._ is ± 5%.

Second, we simulate Ca^2+^ concentrations based on our model, [Ca]_T,model_, which pertains to the maximum amount of Ca^2+^ that can be dissolved from a 10 g L^–1^ PKAT suspension containing 69.9 wt % Ca_3.08_Al_2_(SiO_4_)_0.05_(OH)_11.96_ and 3.2 wt % CaCO_3_:

$${\left[\mathrm{Ca}\right]}_{T,\mathrm{model}}=\frac{c_{\mathrm{katoite}}}{M_{\mathrm{katoite}}}\times 3\times 0.699+\frac{c_{\mathrm{calcite}}}{M_{\mathrm{calcite}}}\times 0.032$$ 11

where *c* is the mass concentration and *M* is the molar mass of Ca_3.08_Al_2_(SiO_4_)_0.05_(OH)_11.96_ and CaCO_3_, respectively. We find that [Ca]_T,model_ matches [Ca]_Tmeas._ within 1.6% exactly at 5 equiv HCl (dashed line in Figure 8), which lends credibility to the model. (Note that [Ca]_T,model_ gradually decreases due to the increasing dilution, as increasing volumes of HCl were added to the suspensions.) At 6 equiv of HCl (pH ≈ 4.3), [Ca]_T,meas_ > [Ca]_T,model_, indicating that C–S–H starts to dissolve in the acidic region, in line with the increase in [Si]_T_ (Figure 5b).

**Figure 9:**
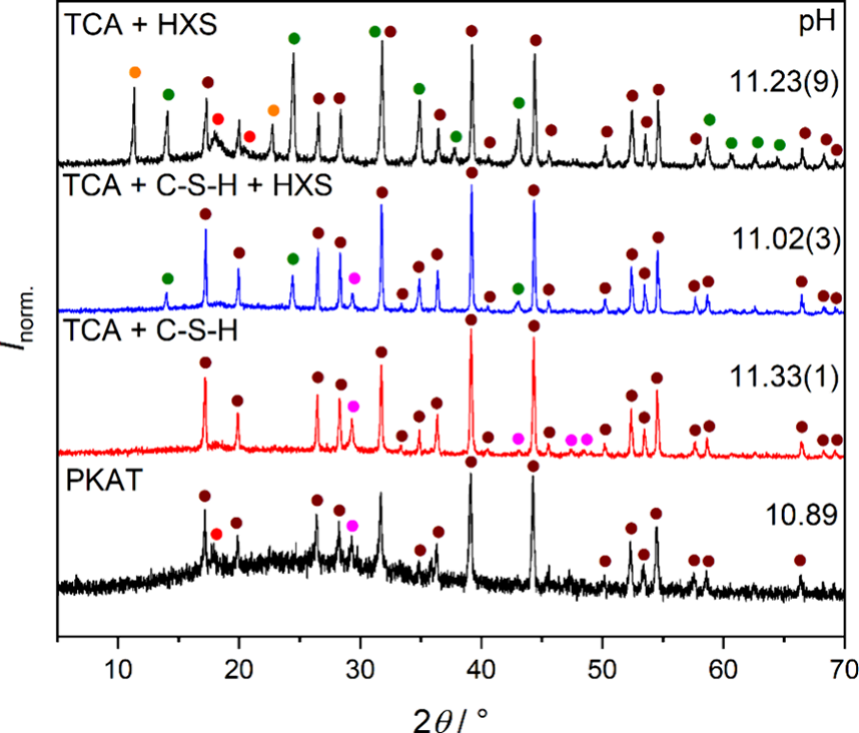
Powder X-ray diffractograms of Ca_3_Al_2_(SiO_4_)_0.9_(OH)_8.4_ (PKAT, nominal composition), 75 wt % Ca_3_Al_2_(OH)_12_ (TCA) + 25 wt % calcium silicate hydrate (C–S–H, Table 1), 75 wt % TCA + 14 wt % C–S–H + 11 wt % hydroxysodalite (HXS, Table 1), as well as 75 wt % TCA + 25 wt % HXS. Solids were obtained from 10 g L^–1^ suspensions neutralized with 2 equiv HCl (*t*_reaction_ = 2 days). Different solid phases are represented by symbols; brown: TCA/PKAT, orange: Cl-LDH, red: gibbsite, magenta: calcite, green: HXS. Diffraction intensities are normalized such that the highest value is unity. Also shown are the pH values of the suspensions before filtration. The standard deviations of three parallel experiments are indicated in parentheses.

Finally, we used this model to reproduce solid-phase transformations for PKAT upon neutralization. To this end, 75 wt % TCA with either 25 wt % as-prepared C–S–H (Table 1), or 25 wt % as-prepared HXS (Table 1), or 14 wt % C–S–H and 11 wt % HXS were mixed, with a final suspension concentration of 10 g L^–1^. 2 equiv HCl was added to these solids, which were filtered after 2 days, and for simplicity, all compositions were regarded as pure TCA for calculation of the acid equivalent. Three parallel experiments were carried out; representative diffractograms and average pH values are shown in Figure 9. As a reference, the diffractogram of PKAT with 2 equiv of HCl is also shown.

We find that, similar to PKAT, there is no LDH formation for TCA + C–S–H and TCA + C–S–H + HXS mixtures, respectively. Strikingly, this suggests that the transformation of TCA to Cl-LDH can be hindered, even by physically mixing TCA with calcium silicate hydrate. In turn, this supports the assumption of as-prepared PKAT being also a mixture of almost pure TCA (Ca_3.08_Al_2_(SiO_4_)_0.05_(OH)_11.96_), C–S–H, and sodium aluminosilicate. Concerning the liquid phase of the three-component mixture before filtration, its pH (11.0) is very close to that of PKAT (10.9). Thus, the model-based mixture represents as-prepared PKAT.

Furthermore, Cl-LDH appears when mixing TCA only with HXS, providing important insight into the influence of minor phases on the formation of Friedel’s salt. That is, the conversion of TCA to Cl-LDH is impaired only when silicates are associated with Ca (and not with Al) atoms. To answer whether this is entirely due to the C–S–H phases acting as seed poison for LDH crystallization and what the exact mechanism of “poisoning” warrants further investigation. In this respect, it is imperative to synthesize katoites with much greater degrees of 4OH^–^/SiO_4_^4–^ substitution and study their neutralization processes. Such experiments are currently in progress.

### Importance of This Study in Bauxite Residue Neutralization

The neutralization properties of a BxR sample are best described by measuring its titration curve.^9,10^ The two most important features of such a curve are the buffering regions in the alkaline range and the acid consumption capacity of the solid.^1,9,10^ The buffering region reflects the type of solid dissolving in that specific pH range, and its length is proportional to the mass concentration of the phase.^1^ Previously, the buffering pH of TCA has been calculated to be pH ≈ 10.^10^ The middle point of the TCA buffering plateau in this work has been found to be ∼11.5 (Figure 5a), which is much higher than pH ≈ 10. One plausible reason for this difference is that the earlier model used tricalcium aluminate, i.e., Ca_3_Al_2_O_6_, for solubility calculations, which has much lower solubility than TCA,^10,12,57^ thereby yielding a lower calculated pH buffer zone. On the other hand, Ca_3_Al_2_O_6_ hydrates rapidly in water;^57^ hence, it is the equilibrium phase in BxR slurries.^1,2,5,9,13^ Further, the model did not account for the formation of Cl-LDH, which has similar solubility as that of TCA, i.e., the pH of both their saturated solutions is 11.83 (infinite dilution).^12^ In turn, this gives rise to similar buffering zones even if all TCA is transformed into Cl-LDH. In this context, PKAT, which does not exhibit LDH formation, can therefore be regarded as a “true” representative of the neutralization behavior of TCA.

Second, the present study provides evidence that the acid consumption of BxR, in which solid alkalinity stems predominantly from TCA or poorly substituted katoite, is always 6 equiv of HCl (or any strong acid), which is independent of Cl-LDH formation. This finding may be translated into actionable insights in the context of on-site BxR neutralization, particularly if the residue is rich in TCA or PKAT: the amount of strong acid required for pH ≈ 8 can be estimated based on the katoite content, whereas attaining a stable pH requires ca. ∼2 days. (Note that these observations are reliable only if there is no cross-reaction between PKAT and other acid-consuming phases.)

However, the presence of any sparingly dissolving amorphous minor phase apparently decreases the acid consumption proportionally to the relative amount of such solids. This highlights the role of the amorphous phases present in the residue, which is estimated to be ∼30%.^1^ In this respect, the formation of amorphous aluminosilicate always precedes various sodalites and cancrinites,^58^ whereas addition of Ca­(OH)_2_ to aid desilication^1,5,9,15^ is likely to promote the precipitation of amorphous C–S–H phases and/or calcium silicates. Indeed, BxR may contain almost 50 wt % calcium silicate (Ca_2_SiO_4_).^59^ Thus, these two solids are conceivable amorphous phases, affecting the neutralization behavior of BxR.

Another area where the outcome of this study may be relevant is the hydration of cementitious materials, e.g., Portland cement, initially governed by the reaction between Ca_3_Al_2_O_6_ and water to form TCA.^17,57,60^ During hydration, OH^–^ and Al­(OH)_4_^–^-containing layered double hydroxides are formed.^17,60^ Eventually, C–S–H becomes the main hydration product of hardened Portland cement paste,^61^ but it can also be introduced to alternative cements prepared using BxR.^4^ This study suggests that the formation of layered double hydroxides upon Ca_3_Al_2_O_6_ hydration might be suppressed in the presence of C–S–H (or calcium silicate).

## Conclusions

In this work, pseudokatoites (PKATs) were prepared by reacting tricalcium aluminate (TCA) with water glass at room temperature. The affinity of silica to be incorporated into the structure of TCA is very limited as Rietveld analysis yielded a formula of Ca_3.08_Al_2_(SiO_4_)_0.05_(OH)_11.96_. On the other hand, the composition of Ca_3_Al_2_(SiO_4_)_0.9_(OH)_8.4_ obtained from ICP-MS showed much higher silicate content. Thus, excess silica present in PKAT must be present as amorphous (nondiffracting) minor calcium silicate hydrate (C–S–H) and sodium aluminosilicate phases.

Upon addition of HCl, TCA, and PKAT, both dissolve but via different mechanisms. TCA transforms to Cl-LDH above 1 equiv of added HCl (or below pH ≈ 11.8), which is indicated by a continuous increase in [Ca]_T_ in the solution phase. Lowering the pH, poorly crystalline gibbsite appears and becomes more prominent with increasing acid equiv, thus decreasing [Al]_T_. As for PKAT, its reaction with HCl is again reflected by the increase in [Ca]_T_; however, it occurs without the Cl-LDH intermediate. That is, minor phases have a crucial role in the neutralization behavior of PKAT, possibly acting as a crystal poison for LDH. Moreover, experiments show that LDH formation is hindered even by physically mixing pure TCA with as-prepared C–S–H.

Furthermore, TCA requires 6 equiv of HCl for its neutralization (pH ≈ 7–8), in excellent agreement with its formula. Conversely, PKAT consumes 5 equiv of acid, indicating that sparingly soluble C–S–H and sodium aluminosilicate decrease the expected acid consumption. This difference can be reproduced by accounting for the presence of these solids via model calculations.

Overall, we find that despite PKAT being essentially identical to TCA, minor phases present (C–S–H in particular) dramatically affect both the acid consumption and the neutralization mechanism of PKAT. We believe that these results help us understand the neutralization reactions of BxR slurries, in particular, the shape and equivalence point of their titration curves.

## Supplementary Material



## Notes

X-ray powder diffractograms of tricalcium aluminate, TCA, hydroxysodalite, and C–S–H together with reference patterns; brief discussion of PKAT syntheses at 95 °C using tricalcium aluminate and corresponding diffractograms; diffractograms of PKATs prepared from TCA at room temperature; thermogravimetric curves of as-prepared TCA and PKAT; scanning electron micrographs of as-prepared KAT (PDF)
