# Supplementary material for: Effect of Amorphous Silicates on the Neutralization of Tricalcium Aluminate Hexahydrate Relevant to Bauxite Residue Treatment
Source: Inorg Chem. 2026 Feb 23;65(9):5178–90. doi: 10.1021/acs.inorgchem.6c00005 (PMC12977044; doi:10.1021/acs.inorgchem.6c00005)
Supplement: Supplementary file 1 [file ic6c00005_si_001.pdf]

Supporting Information

The effect of amorphous silicates on the neutralization of tricalcium aluminate hexahydrate relevant to bauxite residue treatment

Yvette Szabó[a], Meerab Asher[a], Réka Zahorán[a], Judit Papp[a], Dániel Sebők[b], Pál Sipos[a], Márton Szabados[a], Markus Gräfe[c], and Bence Kutus[a]*

[a]Department of Molecular and Analytical Chemistry, University of Szeged, Dóm tér 7–8, H–6720 Szeged, Hungary
[b]Department of Applied and Environmental Chemistry, University of Szeged, Rerrich Béla tér 1, H–6720 Szeged, Hungary
[c]Bauxite Residue R&D Group, Technology Development & Transfer, Emirates Global Aluminium, PO Box 3627, Dubai, United Arab Emirates

** Corresponding author. E-mail address: kutusb@chem.u-szeged.hu (B. Kutus)*

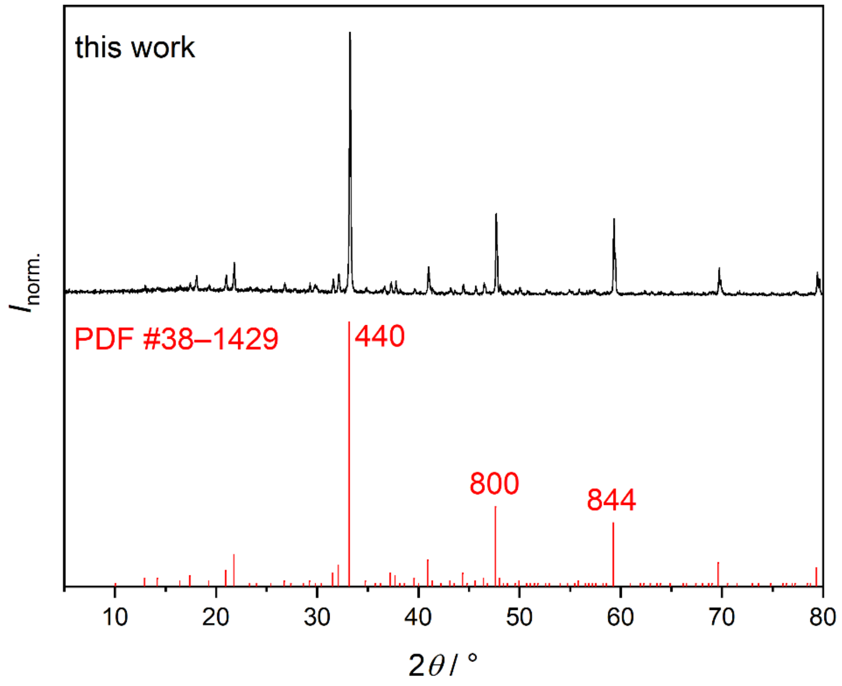

Figure S1 Powder X-ray diffractograms (Cu*Kα*) of tricalcium aluminate, $Ca_3Al_2O_6$, prepared in this work and used as precursor for $Ca_3Al_2(OH)_{12}$ (TCA). Also shown is a literature reference [S1] indicating the Miller indices of the three most intense reflections. Diffraction intensities are normalized such that the highest value is unity.

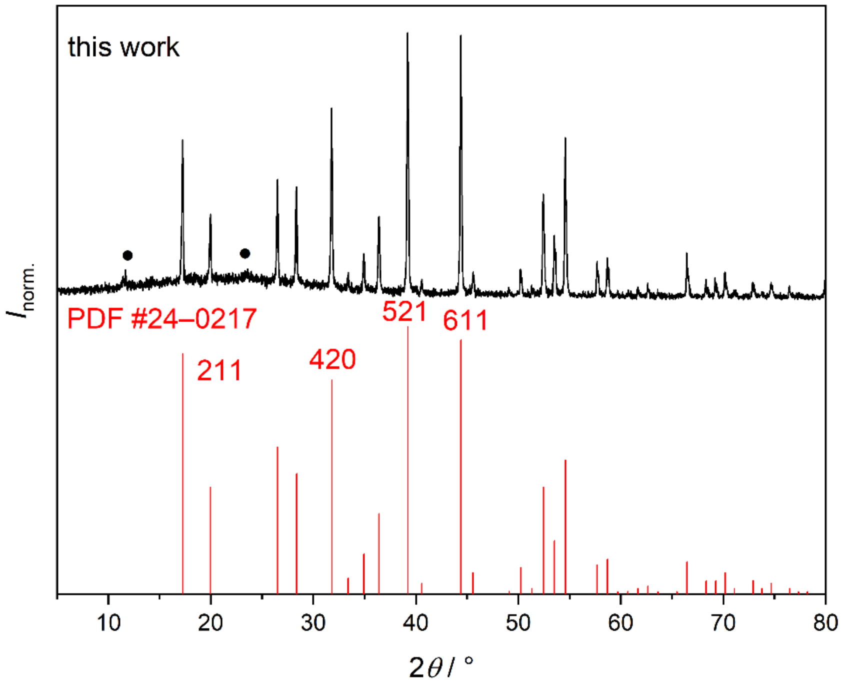

Figure S2 Powder X-ray diffractograms (Cu$K\alpha$) of $Ca_3Al_2(OH)_{12}$ (TCA), prepared in this work. Also shown is a literature reference [S1] indicating the Miller indices of the four most intense reflections. Black symbols represent OH- and CO_3-containing layered double hydroxide (LDH) phases. Diffraction intensities are normalized such that the highest value is unity.

Preparation of KAT by hydrating tricalcium aluminate

We attempted to make a range of katoites (KATs, $x = 0.1$–1) via direct hydration of $Ca_3Al_2O_6$ with water in the presence of either SiO_2 fume or water glass (solid:liquid ratio = 1:5, $t = 2$ days, $T = 95$°C; see Figures S3 and S4). The reference solid TCA shows small-intensity diffractions corresponding possibly to either hemi- ($[Ca_2Al(OH)_6]_2(CO_3)_{0.5}(OH)\cdot 4H_2O$) or monocarboaluminate ($[Ca_2Al(OH)_6]_2(CO_3)\cdot 5H_2O$) LDH phases (OH/$CO_3$-LDH) [S2].

Upon addition of silica fume (Figure S3), a second phase appears, reflected by the shoulders on the right side of the main diffraction peaks of TCA (gray symbols). Based on literature, this phase has been identified as silica-poor KAT with an approximate composition of $Ca_3Al_2(SiO_4)_{0.4}(OH)_{10.4}$ [S3,S4]. Qualitatively, TCA has always longer unit cell lengths than any KAT, hence, the diffraction patterns of the latter show at higher 2θ values [S3–S5]. Upon increasing the silica content in the reaction further, however, the fraction of KAT decreases and TCA remains the dominant phase with a small amount of an amorphous C-S-H phase (blue

symbols) present. In particular, the peak at 29.3° matches with that of as-prepared calcium silicate hydrate (Figure S6) and that of reported in literature [S6]. Using water glass instead of silica resulted in the mixture of unreacted TCA, as well as C-S-H, $Al(OH)_3$ and sodium aluminosilicate phases (Figure S5).

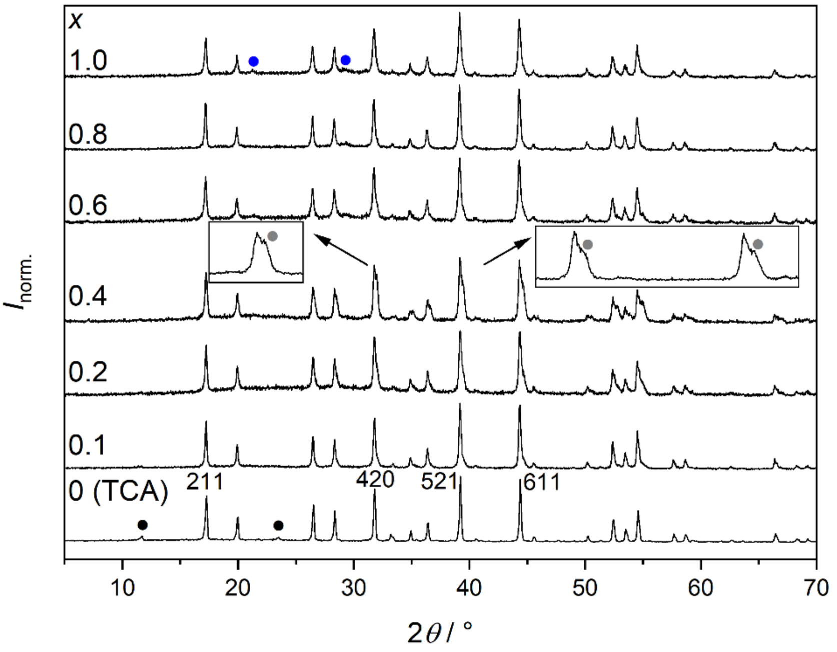

Figure S3 Powder X-ray diffractograms of katoites (KAT), $Ca_3Al_2(SiO_4)_x(OH)_{12-4x}$, prepared by hydrating $Ca_3Al_2O_6$ in the presence of SiO_2 (x = 0–1, $t_{reaction}$ = 2 days, T = 95°C). The nominal degree of substitution, x, refers to the target values. As reference, $Ca_3Al_2(OH)_{12}$ (TCA) and the Miller indices of its four most intense diffraction peaks are shown. Black symbols represent OH/CO_3-LDH phases, whereas blue ones correspond to C-S-H phases. The shoulders labelled with gray symbols in the zoomed regions (30.5–33° and 38–46°) belong to KAT with $x \approx 0.4$. Diffraction intensities are normalized such that the highest value is unity.

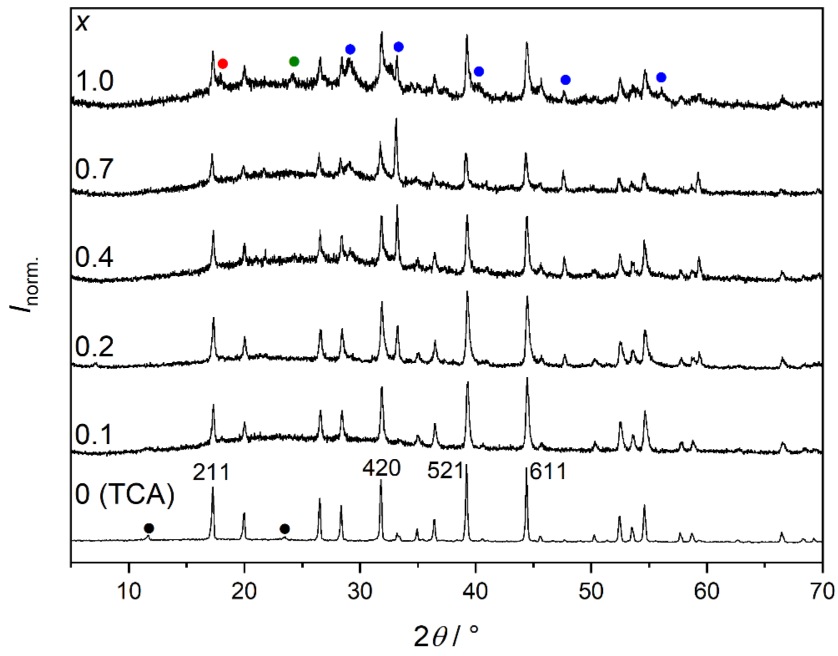

Figure S4 Powder X-ray diffractograms of katoites, $Ca_3Al_2(SiO_4)_x(OH)_{12-4x}$, prepared by hydrating $Ca_3Al_2O_6$ in the presence of Na_2SiO_3 solution (x = 0–1, $t_{reaction}$ = 2 days, T = 95°C). The degree of substitution, x, refers to the target values. As reference, $Ca_3Al_2(OH)_{12}$ (TCA) and the Miller indices of its four most intense diffractions are shown. Different by-products are represented by symbols: OH/OH/CO_3-LDH (black), C-S-H phases (blue), gibbsite (red) and sodium aluminosilicate (green). Diffraction intensities are normalized such that the highest value is unity.

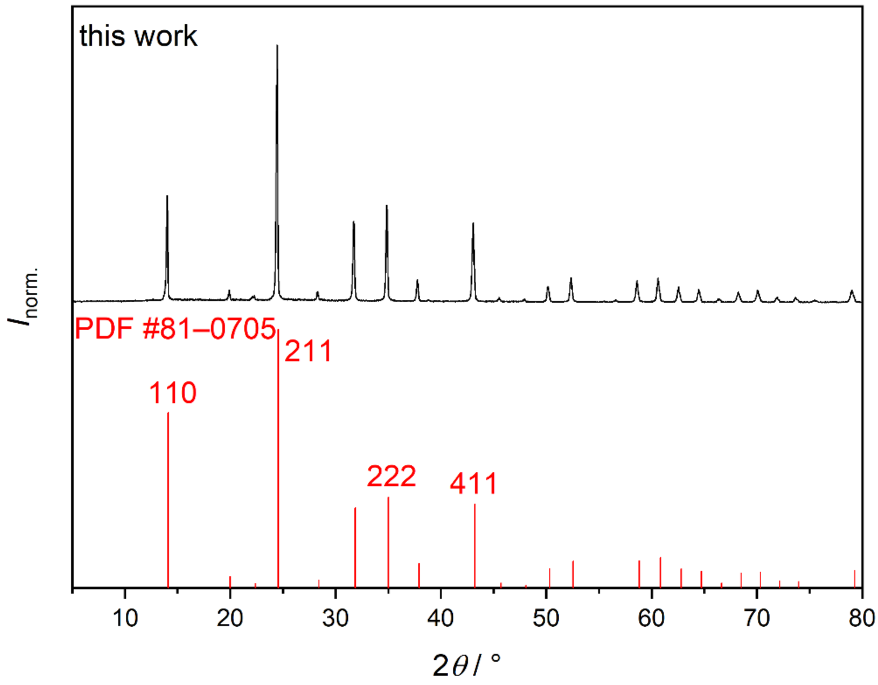

Figure S5 Powder X-ray diffractograms of hydroxysodalite (HXS), prepared in this work. Also shown is a literature reference [S1] indicating the Miller indices of the four most intense reflections. Data are normalized such that the highest value is unity.

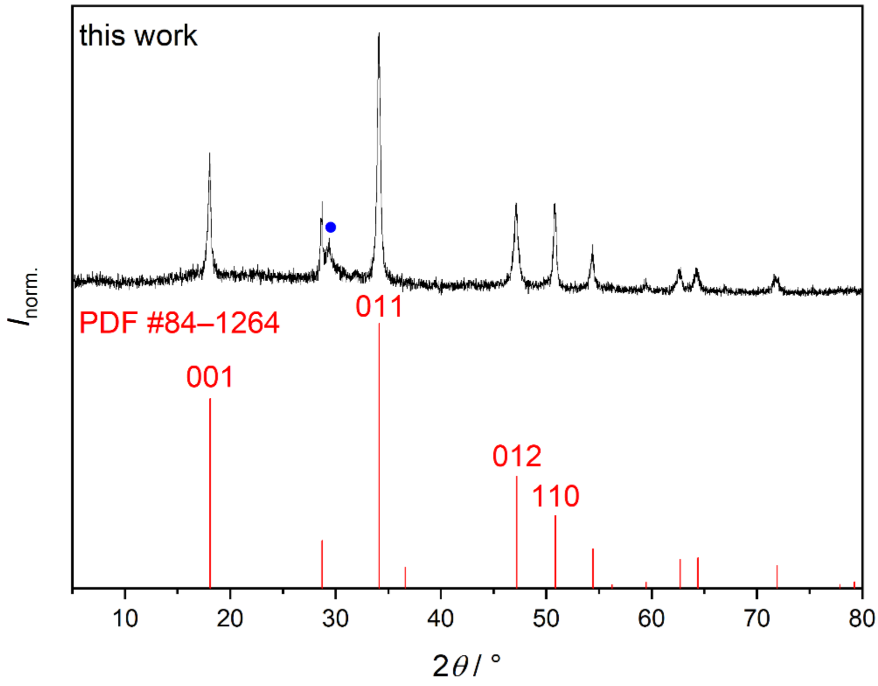

Figure S6 Powder X-ray diffractograms of calcium silicate hydrate (C-S-H, labelled by a blue symbol [S2]), prepared in this work. The obtained solid is dominated by $Ca(OH)_2$, as shown by comparing it with its literature reference [S1]. The Miller indices of the four most intense reflections are also indicated. Diffraction intensities are normalized such that the highest value is unity.

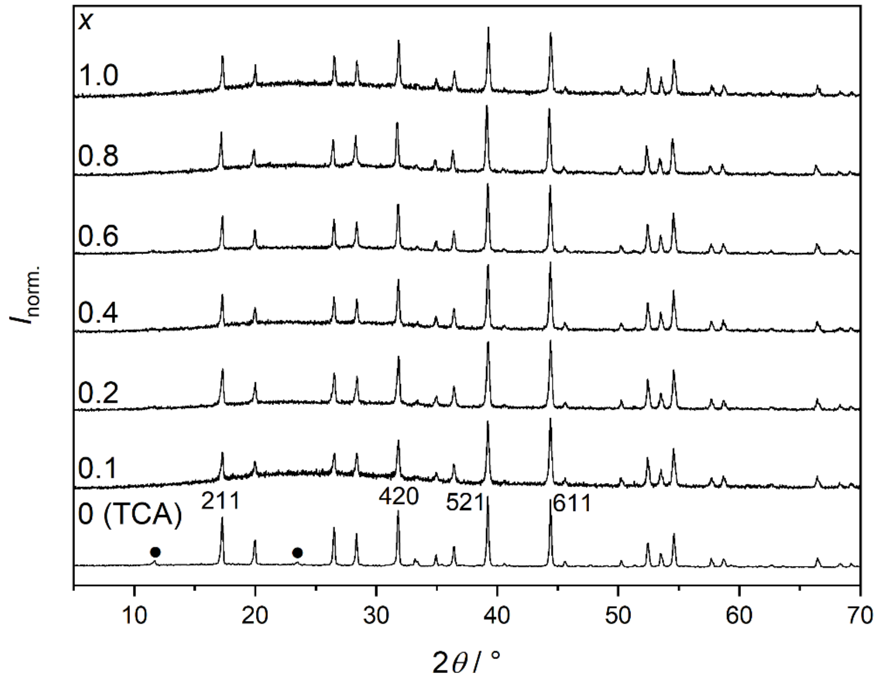

Figure S7 Powder X-ray diffractograms of pseudo-katoites (PKATs), $Ca_3Al_2(SiO_4)_x(OH)_{12-4x}$, prepared by reacting $Ca_3Al_2(OH)_{12}$ (TCA) with Na_2SiO_3 solution (x = 0–1, $t_{reaction}$ = 1 day, r. t.). The degree of substitution, x, refers to the target values. As reference, TCA and the Miller indices of its four most intense diffractions are shown. Black symbols represent CO_3-LDH phases. Diffraction intensities are normalized such that the highest value is unity.

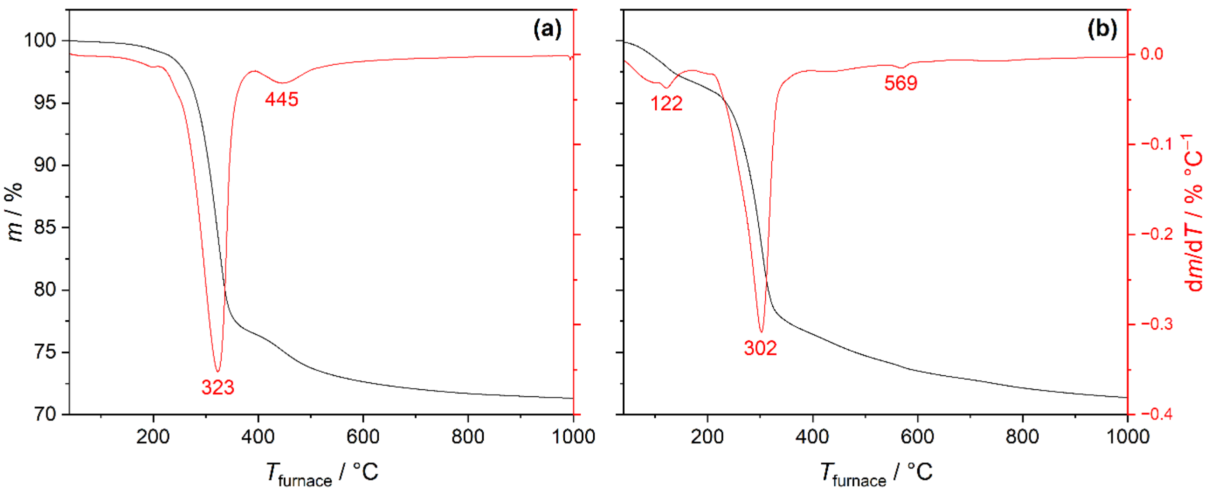

Figure S8 Thermogravimetric analysis of (a) $Ca_3Al_2(OH)_{12}$ (TCA) and (b) $Ca_3Al_2(SiO_4)_{0.9}(OH)_{8.4}$ (PKAT). The left axis corresponds to the mass loss upon heating, while the right axis shows its derivative. Also shown are the characteristic losses, each corresponding to an inflection point on the TG curve.

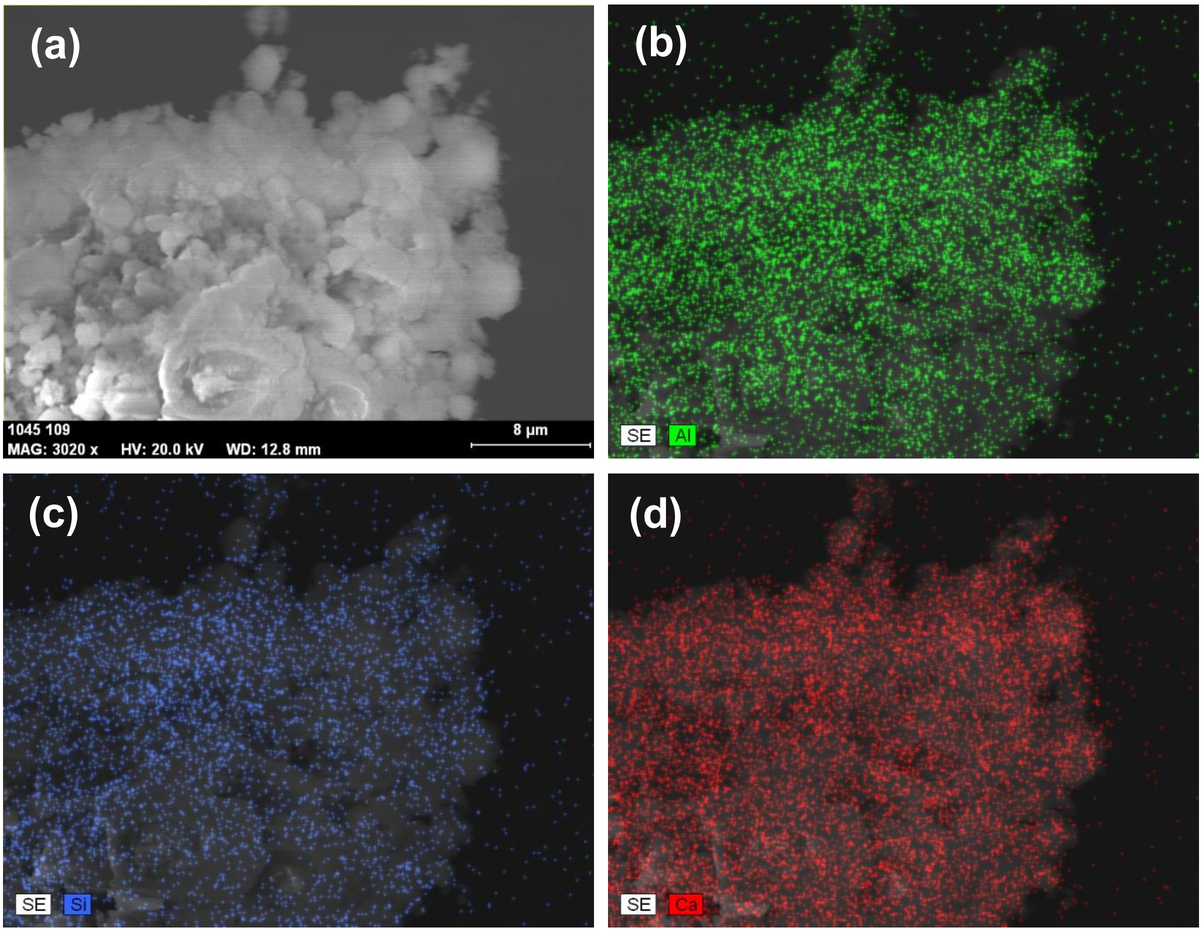

Figure S9 (a) Scanning electron micrographs of $Ca_3Al_2(SiO_4)_{0.9}(OH)_{8.4}$ and elemental distribution for (b) Al, (c) Si, and (d) Ca.

References

[S1] Gates-Rector, S.; T. Blanton. The Powder Diffraction File: A quality materials characterization database. *Powder Diffraction* **2019**, 34, 352–360.

[S2] Baquerizo, L. G.; Matschei, T.; Scrivener, K. L.; Saeidpour, M.; Wadsö, L. Hydration states of AFm cement phases. *Cement and Concrete Research* **2015**, *73*, 143–157.

[S3] Jappy, T. G.; Glasser, F. P. Synthesis and stability of silica-substituted hydrogarnet $Ca_3Al_2Si_{3-x}O_{12-4x}(OH)_{4x}$. *Advances in Cement Research* **1991**, *4*, 1–8.

[S4] Dilnesa, B. Z.; Lothenbach, B.; Renaudin, G.; Wichser, A.; Kulik, D. Synthesis and characterization of hydrogarnet $Ca_3(Al_xFe_{1-x})_2(SiO_4)_y(OH)_{4(3-y)}$. *Cement and Concrete Research* **2014**, *59*, 96–111.

[S5] Kyono, A.; Arora, S. Crystal structure change in grossular–Si–free katoite solid solution: Oxygen position splitting in katoite. *Journal of Mineralogical and Petrological Sciences* **2019**, *114*, 189–200.

[S6] Maddalena, R.; Hall, C.; Hamilton, A. Effect of silica particle size on the formation of calcium silicate hydrate [C-S-H] using thermal analysis. *Thermochimica Acta* **2019**, *672*, 142–149.
